# In Vivo CD8+ T-Cell Suppression of SIV Viremia Is Not Mediated by CTL Clearance of Productively Infected Cells

**DOI:** 10.1371/journal.ppat.1000748

**Published:** 2010-01-29

**Authors:** Joseph K. Wong, Matthew C. Strain, Rodin Porrata, Elizabeth Reay, Sumathi Sankaran-Walters, Caroline C. Ignacio, Theresa Russell, Satish K. Pillai, David J. Looney, Satya Dandekar

**Affiliations:** 1 Department of Medicine, University of California San Francisco, San Francisco, California, United States of America; 2 Department of Medicine, VA San Diego Healthcare System, University of California San Diego, La Jolla, California, United States of America; 3 Department of Physics, University of California Berkeley, Berkeley, California, United States of America; 4 Department of Medical Microbiology and Immunology, University of California Davis, Davis, California, United States of America; NIH/NIAID, United States of America

## Abstract

The CD8+ T-cell is a key mediator of antiviral immunity, potentially contributing to control of pathogenic lentiviral infection through both innate and adaptive mechanisms. We studied viral dynamics during antiretroviral treatment of simian immunodeficiency virus (SIV) infected rhesus macaques following CD8+ T-cell depletion to test the importance of adaptive cytotoxic effects in clearance of cells productively infected with SIV. As previously described, plasma viral load (VL) increased following CD8+ T-cell depletion and was proportional to the magnitude of CD8+ T-cell depletion in the GALT, confirming a direct relationship between CD8+ T-cell loss and viral replication. Surprisingly, first phase plasma virus decay following administration of antiretroviral drugs was not slower in CD8+ T-cell depleted animals compared with controls indicating that the short lifespan of the average productively infected cell is not a reflection of cytotoxic T-lymphocyte (CTL) killing. Our findings support a dominant role for non-cytotoxic effects of CD8+ T-cells on control of pathogenic lentiviral infection and suggest that cytotoxic effects, if present, are limited to early, pre-productive stages of the viral life cycle. These observations have important implications for future strategies to augment immune control of HIV.

## Author Summary

The recognition and elimination of infected host cells by CD8+ T-lymphocytes is held to be a key component of the immune response against viral pathogens. However, this basic tenet of viral immunology may not hold true for HIV and the related SIV. In the current work, we eliminated CD8+ T-cells by treating simian immunodeficiency virus (SIV) infected macaques with a CD8-depleting monoclonal antibody then treated the animals with antiretroviral drugs and measured virus levels. Viral levels fell just as fast for the animals with or without CD8+ T-cells, implying that survival of infected cells producing SIV was not impacted by the presence or absence of CD8+ T-cells. Virus obtained after CD8+ T-cell depletion showed changes in the types of sequences in a viral protein (Nef) that is expressed early after infection of a cell but not in a viral protein (Gag) that is expressed later. These findings suggest CD8+ T-cells have a limited ability to kill cells already expressing SIV but instead may be restricted to non-killing mechanisms or to targeting cells during earlier stages of infection before virus production begins. Understanding and overcoming the factors that prevent CD8+ T-cells from effectively eliminating infected cells producing virus could advance HIV vaccine efforts.

## Introduction

The capacity and limits of host immunity in containing lentiviral infection are fundamental to the understanding of Human Immunodeficiency Virus (HIV) and SIV pathogenesis yet are incompletely understood. Previous studies support effects of host immunity in modulating HIV disease progression [1],[2],[3],[4] and in driving viral evolution and escape. Concurrent with the appearance of HIV specific CD8+ T-cells following either acute HIV or SIV infection, plasma viral load falls abruptly [4], indirectly supporting a role for adaptive, cytotoxic lymphocyte responses in the control of viral replication. However, this evidence is circumstantial and inconclusive, since in most cases of natural infection, several HIV specific immune parameters vary in tandem [3],[5].

The most direct evidence for the antiviral effects of CD8+ T-cells have come from the observation of profound elevations in viral load following the depletion of CD8+ T-cells from SIV infected macaques through the use of anti-CD8 monoclonal antibodies. These studies reveal an approximate ten-fold increase in plasma VL concurrent with CD8+ T-cell depletion [6],[7],[8]. In contrast, similar maneuvers that deplete the CD20+ cells central to humoral immune responses fail to produce comparable effects on viremia [9]. Although a favored interpretation of the CD8+ T-cell depletion experiments attributes the rise in VL to loss of CTL killing [10],[11], this has not been directly demonstrated and the contribution of non-cytotoxic effects of CD8+ T-cells including the production of chemokines that block new infectious events or the elaboration of soluble factors that attenuate viral production from infected cells remain as possible alternative mechanisms [7],[12].

In classic studies of viral dynamics performed by perturbing the VL steady state using antiretroviral drugs that inhibit HIV replication, cell free virus and the infected cells producing HIV were shown to have a very short lifespan [13],[14],[15]. While the models used to explain these dynamics invoke clearance and death of productively infected cells with a half-life of only a day [16], the mechanism responsible for this rapid elimination has yet to be elucidated.

In this study, we measured VL decay during the initiation of antiretroviral therapy in SIV-infected macaques with or without depletion of CD8+ T-cells to assess whether the rise in VL upon CD8+ T-cell depletion was accompanied by an increase in the life span of productively infected cells and, conversely, to determine whether CTL killing is responsible for the short half-life of productively infected cells *in vivo*. Indirect evidence for immune selective pressure by CTL was assessed by comparing sequence variation in a representative early (*nef*) and late (*gag*) viral genes from samples collected immediately before and right after CD8+ T-cell depletion.

## Methods

### Ethics statement

All animals were handled in strict accordance with good animal practice as defined by the relevant national and local animal welfare bodies and all animal work was approved by the UC Davis Institutional Animal Care and Use Committee (IACUC).

### Infection of rhesus macaques and overview of study

**Figure 1:**
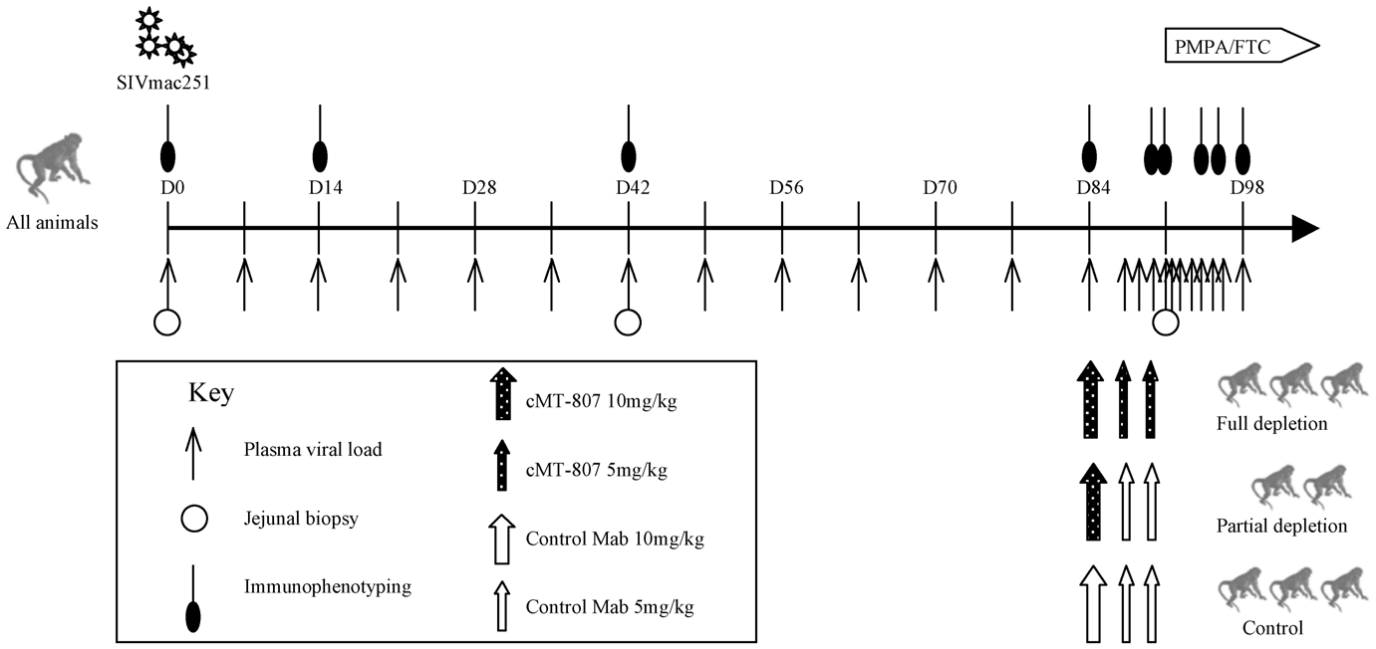
Timeline of experiments. Plasma SIV RNA levels, immunophenotyping, GI biopsy and antibody treatment according to treatment group is shown. D = day post infection. Treatment phase with PMPA/FTC is indicated by the horizontal block-arrow in the right upper hand corner.

Animal studies were conducted in accordance with UC Davis IACUC approved protocols at the California National Primate Center. 8 healthy junvenile rhesus macaques were infected intravenously with 1000 TCID of SIVmac251 at time zero (D0). Viral load testing and immunophenotyping were performed as shown (Figure 1). At week 12 (D84), animals received mAb cMT-807, control antibody or no mAb. At week 13 (D91), animals were started on combination antitretroviral therapy consisting of PMPA (30 mg/Kg per day) and FTC (8 mg/Kg per day) given intramuscularly (IM) daily.

### CD8+ T-cell depletion

12 weeks post-infection (D84), 3 animals received full depleting doses of monoclonal antibody (mAb) cMT-807, a mouse/human chimeric monoclonal antibody, administered IM (Centocor, Horsham, PA) on D84, D87 and D91, 2 animals received partial depleting treatment with a single dose of cMT-807 followed by control antibody and 3 control animals received a series of control antibody injections (1) or no antibody treatment (2) (Figure 1).

### Viral load testing and immuno-phenotyping

VL testing was performed on peripheral blood plasma using a real time PCR assay detecting a sequence in SIV *gag* as previously described [17],[18]. Immuno-phenotyping was performed as previously described. To assess whether the effect of epitope masking by cMT-807 might contribute to the measured depletion of CD8+ cells, a second staining mAb CD25 (DAKO, Carpinteria, CA) was used to corroborate the depletion achieved in two CD8-depleted animals and changes in proportions of CD4/CD8 double negative populations before and after treatment with cMT-807 were sought [7].

### Endoscopy and gut biopsy

Jejunal pinch biopsy samples were incubated in RPMI 1640 (Gibco/Invitrogen, Carlsbad, CA) and collagenase (Sigma, St. Louis, MO) at 37°C and rapidly shaken for 45 minutes and then subjected to Percoll (Sigma, St. Louis, MO) density gradient centrifugation to enrich for T-cells and eliminate tissue debris [19]. Cells were then washed with phosphate buffered saline (PBS) (Gibco/Invitrogen, Carlsbad, CA) and allowed to equilibrate over night at 37°C and 5% CO_2_ in complete RPMI 1640 (containing 10% fetal calf sera, penicillin and streptomyicin). PBMC were isolated by density centrifugation over Lymphocyte Separation Media (LSM) (Organon-Technica, Durham, NC). Aliquots of freshly isolated cells were then stained with fluorescently labeled antibodies for flow cytometric analysis.

### Antiretroviral therapy

On D91, all animals initiated daily intramuscular injections of 30mg/kg/day of PMPA (Gilead, Foster City, CA) and 8mg/kg/day of FTC (Gilead, Foster City, CA) [19] until they were euthanized and necropsied at week 15.

### Modeling viral dynamics

We based our estimates for clearance of productively infected cells on first-phase plasma virus clearance rates as proposed by Ho et al and Wei et al [13],[15]. The model employed was a basic model proposed by Perelson et al [14].

We used Maximum likelihood fits of our measured data during the first week on ART to estimate the clearance rate “δ” [14] and the calculated half life (t1/2) of productively infected cells: *t*1/2 = ln(2)/δ. The baseline timepoint was excluded from these fits to exclude the effect of the rapid elimination of cell-free plasma virus from the blood compartment (*t*1/2<1 hr.) on viral decay. We chose to limit the data for the inclusion in calculation of the first phase decay to those prior to 7 days (between 0.5 to 5 days inclusive) in order to avoid any possibility that recovery of CD8+ T-cells could affect the decay estimates. For the same reason, we have not attempted to model second phase decay. The difference in cell death rates between animals with and without depletion of CD8+ T-cells provides a measure of the effect that CTL killing has on shortening the lifespan of productively infected cells. Correlation between measures of CD8 T-cell depletion and either viral load rebound rate or death rate of productively infected cells (δ), was assessed by a Spearman rank-correlation test.

### Sequencing and sequence analysis of partial *gag* and *nef*

RNA was extracted from 140µl of plasma using the Qiagen QIAamp Viral RNA Kit according to the manufacturer's protocol. cDNA was prepared using random decamers and Ambion RETROscript™ Kit (Ambion, Austin TX). Quadruplicate nested PCRs were carried out for *nef* using primers SIV nef5′out (TGACCTACCTACAATATGGGTG) and SIV nef3′out (TCCCCTTGTGGAAAGTCCCTGCT) and SIV nef5′in (CGTGGRGAGACTTATGGGAGACT) and SIV nef3′in (AAGGCCTCTTGCGGTTAGCCTTC). For the *gag* region, quadruplicate PCRs were carried out using primers SIVgag1151F (AGGAACCAACCACGACGGAG) and SIVgag2445R (AAAGGGATTGGCACTGGTGCGAGG). SuperTaq™ from Ambion (Ambion, Austin, TX) was used for all the PCR reactions. Products were proportionately pooled then cloned using the TOPO TA cloning kit. The *gag* PCR product was gel purified using Qiagen QIAex Gel Extraction Kit prior to being used for cloning. Clones were picked and plasmids prepared using the Qiagen Plasmid Mini Kit (Qiagen, Chatsworth CA) according to the manufacturer's protocol. Plasmids were sequenced using SIVgag1151F and SIVGag1826R (CCTGGCACTACTTCTGCTCC) as sequencing primers for *gag* and SIV nef5′in and SIV nef3′in for *nef*. Big Dye Terminator v3.1 (Applied Biosystems, Foster City, CA) sequencing mix. Sequences were aligned and edited using clustalW as executed in Sequencher and and subsequent manual editing was performed in Se-Al. Estimates of genetic diversity were calculated using Dnadist within the PHYLIP 3.6 software suite [20]. Selection intensity and dN/dS ratios were evaluated using the Synonymous-Nonsynonymous Analysis Program (SNAP), based on the methodology of Nei and Gojobori [21]. A Fisher's Exact test was used to compare site-specifc amino acid composition between timepoints. Sequences are available through GenBank, accession numbers GU366223 to GU366660.

## Results

### Dynamics of acute SIV infection and establishment of cellular and viral load steady state

The SIVmac251 infection of rhesus macaques was chosen as the experimental model because viral dynamics studies in this model of AIDS have been well described [22] and the monoclonal antibody cMT-807 is effective in eliminating CD8+ T-cells in rhesus macaques [6],[23]. The overall experimental plan is shown in Figure 1. All animals were infected with 1000 TCID_50_ of SIVmac251 by intravenous injection and were longitudinally monitored as shown. At Week 12 (D84) post infection, 3 animals were assigned to the “full depletion group” and received 3 doses of cMT-807 at approximately twice the dosage previously used by Schmitz et al [6] in order to achieve more sustained CD8+ T-cell depletion, 2 animals received only a single dose of cMT-807 (partial treatment group) and 1 control animal received three doses of an isotypic control antibody while 2 control animals received no control antibody injections [6].

### Viral infection phase

**Figure 2:**
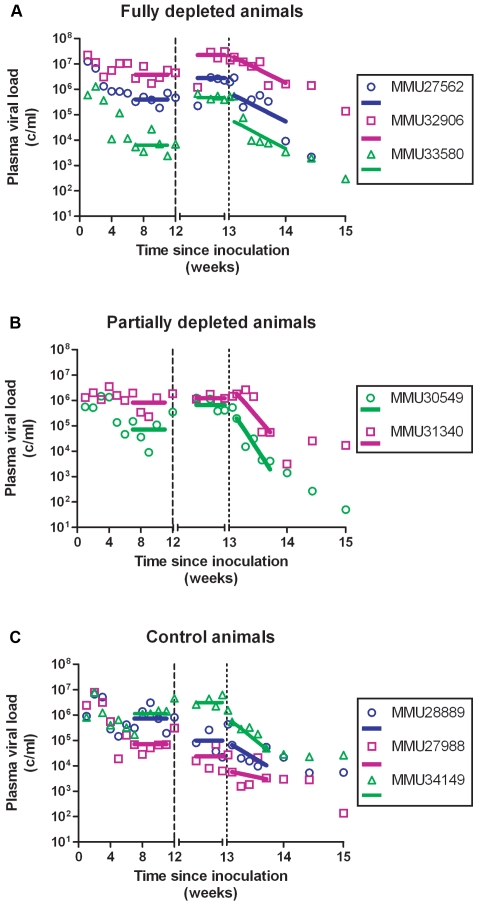
Plasma SIV RNA prior to, following treatment with cMT-807 antibody and over the first week after starting PMPA/FTC. Data shown are grouped according to treatment group with fit lines for the period pre cMT-807 administration, during cMT-807 administration and during antiretroviral therapy shown as the solid line segments. Note that for the tick marks on the X-axis prior to Day 84 (Week 12) is by 21 day (3 week) increments while after Day 84, the timescale is in 4 day increments.

Animals exhibited high plasma viral loads in the first weeks following infection that peaked prior to D28 and declined to a “steady state” set point determined by viral and host factors including host immune responses [3],[4],[22]. The overall pattern appeared consistent with previously published data following acute infection with either SIV or HIV (Figure S1). VL set points prior to D84 were estimated to be 3.8 to 6.6 logs for the 8 animals (Figure 2 and Table 1).

**Table 1:** Summary of viral load dynamics and correlation to CD8 T-cell depletion.

Animal	Peak viral load	Set point (week 7–12)	After Ab treatment	Exponential Fit Parameter (w12–13)**	Half-life Est (d)*	Viral load drop in 1st week of ART	Viral load drop in 2nd week	%CD8 cells in peripheral blood at week 13	% CD8 cells in Lamina Propria at week 13
**MMU27562 (Full)**	7.1	5.6	6.4†	0.32	1.8 (0.8,3.5)	2.2	>3.0	0	11.1
**MMU32906 (Full)**	7.5	6.6	7.4†	0.35	1.8 (0.9,3.5)	0.9	1.9	0	18.9
**MMU33580 (Full)**	6.1	3.8	5.6†	0.55	0.6 (0.5,1.0)	2.2	3.2	0.65	32.5
**MMU30549 (Partial)**	6.2	4.9	5.7†	0.0021	0.7 (0.5,1.1)	2.7	4.1	13.2	48.5
**MMU31340 (Partial)**	6.5	5.9	6.1	−.070	0.8 (0.5,2.2)	2.6	1.8	1.81	52.1
**MMU28889 (control)**	6.8	5.9	5.0	−.448	1.5 (0.6, 2.9)	0.5	1.1	19	52.3
**MMU27988 (control)**	6.9	4.9	4.4	−.419	4.3 (1.0, ∞)	0.7	2.0	46.1	61.3
**MMU34149 (control)**	6.9	6.1	6.6	0.0052	1.1 (0.9,1.8)	2.1	2.1	19	33.1

There were no significant differences between the depleted and control animals in peak viral load, set point, or SIV RNA half-life on antiretroviral therapy.

Viral load set points were computed by averaging all available logarithmic viral loads for each animal between weeks 7 and 12 of the study. After Ab treatment VL were computed by averaging D89, D90, D91 VL. The exponential fit parameter (week 12–13) is the slope of the rise in log viral load during the depletion phase of study. The half-life of productively infected cells was estimated from plasma decay during the first week on ART.

**: Rise in viral load during depletion phase was significantly correlated with CD8% in peripheral blood and lamina propria. Spearman Rank Correlation p≤0.04 and p≤0.007 respectively.

*: Half life of productively infected cells was not correlated with CD8%/depletion.

**†:** Viral load increased significantly following administration of antibody (p<0.05 by Wilcoxon test).

**Figure 3:**
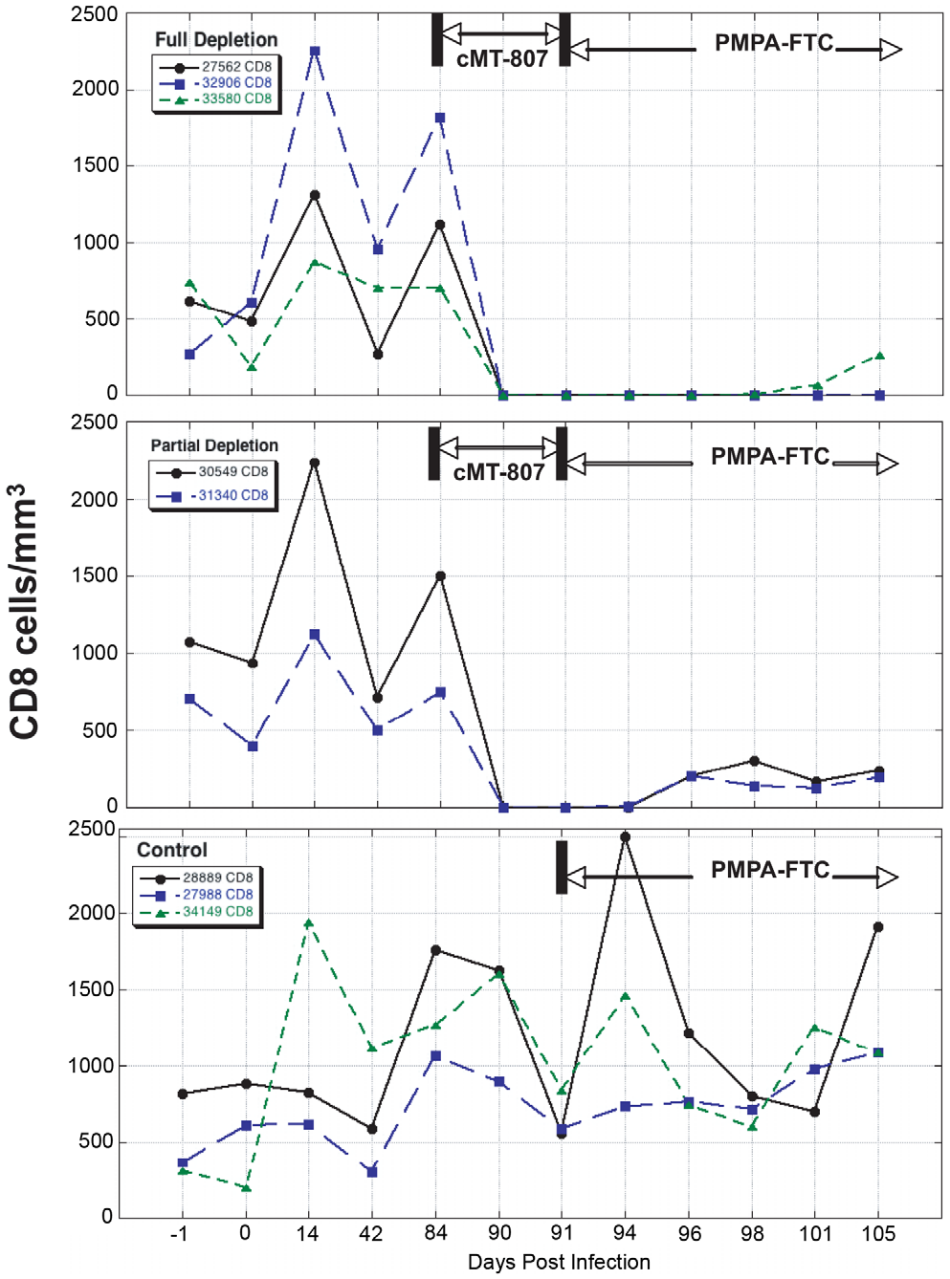
CD8 T-cell concentrations before, following treatment with cMT-807 antibody and after starting PMPA/FTC. Results are displayed separately for full depletion, partial depletion and control animals. Time scales differ for each epoch shown and the x-axes are not to scale.

**Figure 4:**
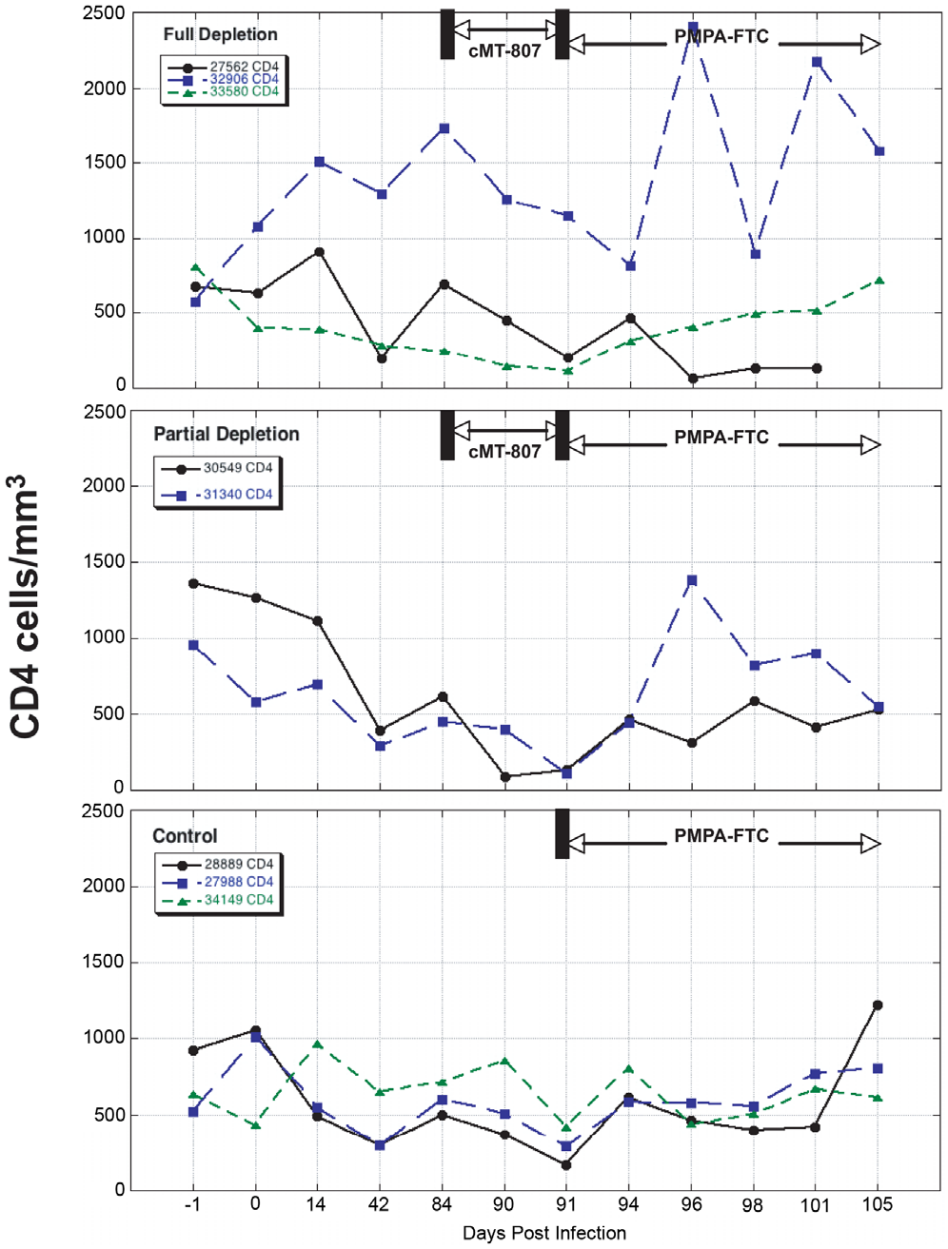
CD4 T-cell concentrations before, following treatment with cMT-807 antibody and after starting PMPA/FTC. Results are displayed separately for full depletion, partial depletion and control animals. Time scales differ for each epoch shown and the x-axes are not drawn to scale.

After 12 weeks of SIV infection, CD4 T-cell counts in whole blood declined from a median of 807 to 693 cells/mm^3^ and CD8+ T-cells rose from 612 to 1247 cells/mm^3^ (Figures 3, 4). There were no significant differences between control and study animals in set point VL, CD4% and CD8% prior to administration of the CD8+ T-cell depleting antibody (Table 1). To the degree that these parameters reflected the equilibrium between pathologic effects of viral replication and effects of host immune response, the animals in the control and treatment arms of the study appeared comparable.

### CD8+ T-cell depletion phase

Frequencies of CD8+ T-cells dropped precipitously with administration of cMT-807 in the full depletion animals, reaching undetectable levels by D91 in all 3 animals (Figure 3). This was accompanied by a 0.7 to 1.9 log_10_ rise in viral loads that appeared sustained, representing a new pseudo-steady state as previously described [6],[7] (Figure 2A). In the partial depletion animals, CD8+ T-cells fell initially but became detectable again by D96 (Figure 3). One partial depletion animal experienced an increase in viral load approximately 1 log above pre-antibody administration-set point at D91 (week 13) (Figure 2B). In the second partially depleted animal, plasma viral loads were unchanged between D84 (week 12) and D91 (week 13) timepoints. No consistent change in CD8+ T-cell counts was experienced by any of the three control animals including the animal receiving the isotype control antibody. CD4 numbers in the treated groups showed a mild gradual decease following administration of cMT-807 but this was also seen to a lesser extent in the control group (Figure 4).

To exclude epitope masking as a cause for the absence of detectable CD8+ T-cells in the peripheral blood by flow cytometry, data were examined for levels of CD3+ CD4/CD8 double negative cells pre and post antibody administration for each CD8+ T- cell-depleted animals as previously performed by Jin et al [7] and independent FACS was performed with DAKO clone DK25 antibody in 2 full depletion animals with sufficient sample for parallel analysis. CD3+ CD4/CD8 double-negative cells were not more frequent after CD8+ T-cell depletion nor were CD8+ T-cells detectable using the second staining antibody (data not shown).

While depletion of CD8+ T-cells in peripheral blood appeared complete in the “full depletion” group of animals, CD8+ T-cell depletion in GALT at week 13 (D91) was less extensive but nonetheless reflected a median reduction of 66% in the animals that received the full depletion protocol. In contrast, median reduction in CD8% was 30% in partial depletion animals and 0% in control animals. These findings are in keeping with the experience of other investigators that appear to confirm extensive depletion of CD8+ T-cells in peripheral blood and central lymphoid tissues, while CD8+ T-cell depletion in the GALT can be substantial but less complete.

### Correlation of CD8+ T-cell depletion in GALT with the increase in plasma VL

**Figure 5:**
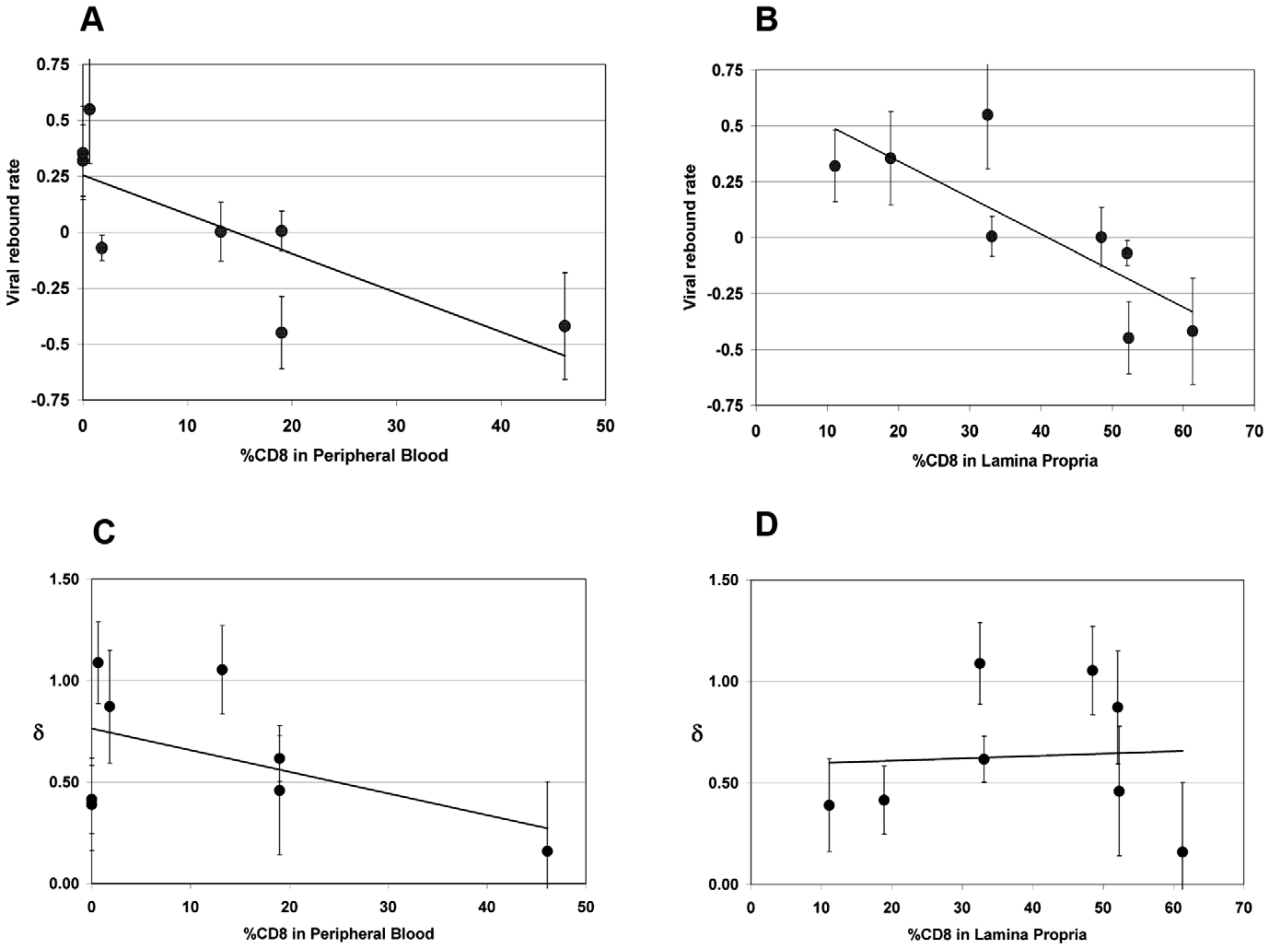
Correlation between viral rebound rate and death rate δ and % CD8 remaining in peripheral blood (PBMC) and lamina propria lymphocytes (LPL). Error bars are shown for estimates. Significant negative correlations were found for viral rebound rates and CD8% in PBMC (5A, Spearman r −0.76, p = 0.036,) and CD8% in LPL (5B, Spearman r −0.88, p = 0.0072,) but not for death rate of productively infected cells, δ, and CD8% in either PBMC (5C, Spearman r = −.12, p = 0.79) or LPL (5D, Spearman r = 0.07, p = 0.88). The fit lines shown are simple regression lines and do not directly correspond to the non-parametric, Spearman correlation.

We exploited variability in CD8+ T-cell depletion in GALT resulting from our use of complete and partial depletion protocols and from biologic variability between animals as a way to gauge a “dose response” between CD8+ T-cell depletion and extent of viral load increase between D84 and D91. The residual CD8% (reflecting extent of depletion) in both peripheral blood and lamina propria lymphocytes (LPL) inversely correlated with the rise in VL observed during treatment with cMT-807 (Spearman r = −0.759, p = 0.036 and r = −0.881, p = 0.0072 respectively), as represented by the slope of the exponential rise in VL between D84 and D91 (viral rebound rate) (Table 1, Figure 5A,B). Because most SIV (and HIV) infection events are thought to take place in lymphoid tissues, the stronger correlation between CD8% of LPL and viral rebound is not surprising. These observations are consistent with a direct effect of CD8 cells in controlling HIV replication but they do not distinguish antiviral effects based on preventing new infection, CTL killing of productively infected cells or inhibition of viral transcription.

### Decay in plasma viremia with combination antiretroviral therapy

At Week 13 (D91), all animals received (30 mg/Kg) PMPA and (8 mg/Kg) FTC after establishment of the new VL steady state resulting from CD8+ T-cell depletion. Previous studies have established that this drug combination results in potent suppression of viral replication in SIV infected macaques as reflected in the prompt fall in plasma viremia (Figure 2). First phase plasma viral decay computed from plasma RNA measurements following initiation of PMPA/FTC was used to estimate t1/2 of productively infected cells. We used data between 0.5 and 5 days after starting antiretroviral drugs to calculate the first phase decay of plasma viremia to allow for a pharmacologic delay and to avoid potential confounding effects from CD8+ T-cell recovery however, using data including day 7 or day 10 did not change the overall observation of very similar decay characteristics between groups. The median calculated half-life for each of the three groups (Table 1) approximated the half-lives of 0.7 to 1.4 days previously reported by Nowak [22]. Unlike the significant correlation between the slope of increase in VL with extent of CD8+ T-cell depletion in blood and LPL, no similar correlation was found between extent of CD8+ T-cell depletion and clearance of productively infected cells, δ (Figure 5C, 5D). Similarly, half-life was not correlated with CD4% or VL prior to antiretroviral therapy (data not shown).

### Evolution of SIV *gag* and *nef* before and after CD8+ T-cell depletion

As an independent probe for potential suppressive effects of CTL on SIV infected cells, we next examined changes in the distribution of viral variants reflected in clonal *gag* sequences (as a representative “late” structural gene) and clonal *nef* sequences (representing early, regulatory and accessory genes). Others have demonstrated that the pace of evolution of SIV immune escape epitopes in macaques following acute infection is altered by CD8+ T-cell depletion [24]. If the approximate 1 log rise in viral load was related to removal of suppressive effects of CTL clones targeting specific SIV epitopes in either Gag or Nef, we hypothesized that sequences would show evidence of positive selection (typically associated with CTL pressure) and a change in distribution of viral variants would occur after CD8+ T-cell depletion. We analyzed partial amino acid sequences of Gag and Nef that were comparable in length from CD8+ T-cell depleted and non-depleted control animals.

**Figure 6:**
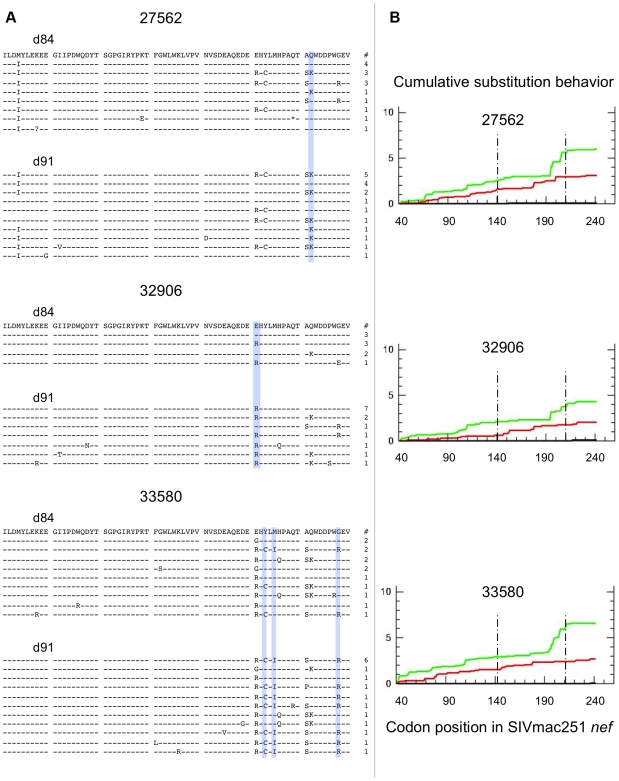
SIV *nef* evolution in CD8-depleted animals. Data from 3 fully depleted animals are shown. A) Sequence alignments of SIVmac251 Nef region demonstrating greatest diversity and evolution during the depletion period (amino acids 141–210). Dashes indicate identity to SIVmac251 inoculum consensus sequence, asterisks indicate premature stop codons, and question marks represent unresolvable sites (due to nucleotide ambiguity). Blue bars highlight positions with statistically significant differences in amino acid distribution between day 84 and day 91 (based on Fisher's Exact, p<0.05). Numbers of sampled clones associated with each predicted protein sequence are listed on right. B) Cumulative behavior of synonymous and nonsynonymous substitutions across *nef* sequence. Green lines = nonsynonymous mutations, red lines = synonymous substitutions; area between dashed lines represents region displayed in panel A.

**Figure 7:**
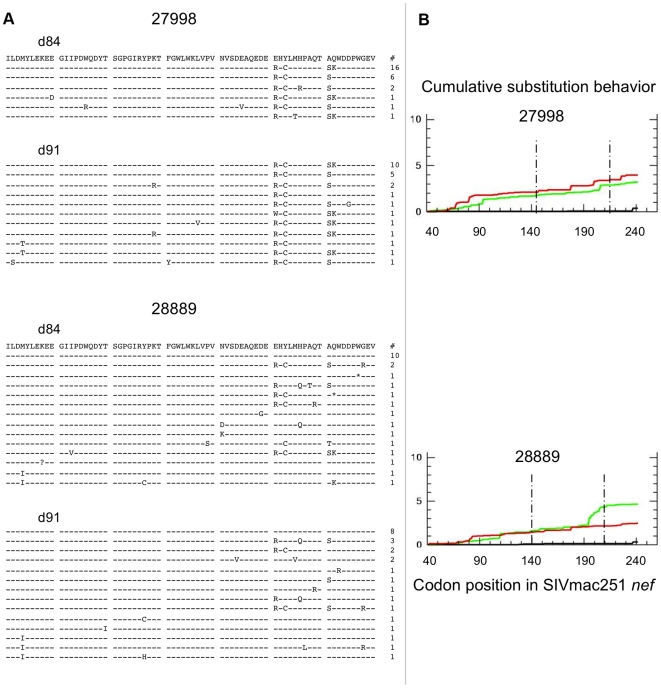
SIV *nef* evolution in control animals. Data from 2 non-depleted control animals are shown. A) Sequence alignments of SIVmac251 Nef region demonstrating greatest diversity and evolution in depleted animals (amino acids 141–210). Dashes indicate identity to SIVmac251 inoculum consensus sequence, asterisks indicate premature stop codons, and question marks represent unresolvable sites (due to nucleotide ambiguity). There were no positions with statistically significant differences in amino acid distribution between day 84 and day 91 (based on Fisher's Exact test). Numbers of sampled clones associated with each predicted protein sequence listed on right. B) Cumulative behavior of synonymous and nonsynonymous substitutions across *nef* sequence. Green lines = nonsynonymous mutations, red lines = synonymous substitutions; area between dashed lines represents region displayed in panel A.

The distribution of Gag sequence variants remained consistent pre and post CD8+ T-cell depletion for all animals (Figure S2). In contrast, several positions in Nef exhibited statistically significant differences in amino acid composition, possibly reflecting release from Nef-specific CTL pressure during the CD8+ T-cell depletion period (Figure 6A). Accordingly, Nef sequences from control animals failed to show any significant redistribution of variants during this period (Figure 7A). An excess of nonsynonymous over synonymous nucleotide substitution was observed across regions of *nef*, in all depleted animals (Figure 6B) and 1 of 2 control animals (Figure 7B), indicative of positive selection consistent with positive or diversifying selection typically associated with CTL pressure. Ratios of nonsynonymous to synonymous substitution (dN/dS) (Figures 6B, 7B and S3) and nucleotide sequence diversity (as represented by pairwise genetic distance) were consistently higher in *nef* clones than in *gag* clones both pre and post CD8+ T-cell depletion (data not shown). We did not have sufficient viable cell samples to directly test for the presence or absence of epitope specific CTL clones to explain the change in distribution of viral variants following CD8+ T-cell depletion. Nevertheless, although stronger functional constraints on Gag may play a role, these sequence findings are compatible with CTL activity disproportionately targeting Nef and are consistent with the observation of O'Connor that CTL driven viral evolution may be greater for early viral genes such as *tat* and *nef*.

## Discussion

The limited success of recent HIV vaccine trials designed to elicit CTL responses to protect at risk subjects from infection underscores the need to reexamine the relative roles of adaptive and innate immunity in the control of HIV infection [25]. The current study was designed to investigate how CD8+ T-cells work to contain pathogenic lentiviral infection *in vivo* by examining changes to the lifespan of productively infected cells inferred from viral dynamics during antiretroviral therapy with or without CD8+ T-cell depletion using the anti-CD8 monoclonal antibody cMT-807. While our data confirmed the importance of CD8+ T-cells in viral control by demonstrating a rapid increase in VL resulting from CD8+ T-cell elimination, they unexpectedly revealed that the rate of clearance of productively infected cells is independent of the extent of CD8+ T-cell depletion. This latter finding challenges the conventional view that the principal contribution of CD8+ T-cells to antiviral immunity is through their function as CTL's that recognize and kill productively infected cells. The suppressive effects of CD8+ T-cells may instead be exerted through non-cytotoxic effects such as those affecting viral expression/production (as might be mediated by the non-cytotoxic antiviral factor described by Walker [26],[27]) or infectivity (such as chemokines that block infection [28]). Alternatively, CD8+ T-cells may suppress viral replication via CTL killing of infected cells but this may be confined to a narrow window in the pre-productive stages of infection [29]. The study by Klatt and colleagues published in this issue of *PLoS Pathogens*, using a different experimental protocol to deplete CD8+ T-cells reached similar conclusions [30].

We confirmed earlier studies reporting a rapid rise in VL following administration of cMT-807 coincident with elimination of CD8+ T-cells. The CD8+ T-cell depletion maneuver could result in several indirect effects that might influence viral replication that deserve consideration. Homeostatic mechanisms that regulate total T-lymphocyte number could result in compensatory increases in CD4+ T-cell numbers. Alternatively, administration of large doses of exogenous antibody with massive cellular depletion could result in generalized T-cell activation and could increase the infectability of target cells without increasing absolute CD4+ T-cell numbers. Earlier studies observed that treatment with anti-CD8 antibodies had little effect on the availability of total CD4 T-cells [6],[7]. We observed moderate reductions in CD4+ T-cell counts in all 3 groups rather than increases in CD4+ T-cell numbers. Finally, Okoye recently demonstrated that the heightened tempo of SIV infection following CD8+ T-cell depletion occurred independently of increases in either absolute CD4+ T-cell number or their activation status [31]. Moreover, the finding that there was a strong correlation between the extent of CD8+ T-cell depletion at the putative sites of SIV replication in GALT and the rate of rise in VL after cMT-807 administration provides additional support that it is CD8+ T-cell depletion and not administration of exogenous antibody per se that was responsible for heightened viral replication. Finally, cMT-807 used in these experiments targets the CD8 molecule expressed on both CD8+ T-cells and some NK cells (CD8+, CD3−). Thus, the viral load increases following treatment with cMT-807 could have resulted from both CD8+ T-cell and partial NK cell depletion.

The observation that first phase decay of plasma viremia during treatment with potent inhibitors of viral replication is not affected by depletion of CD8+ T-cells suggests that CTL killing is not necessary to effect the rapid turnover of productively infected cells seen in this and many previous viral dynamic studies (nor does it seem that CD8+, CD3− NK cells would be required although this was not specifically evaluated). It should be noted that the productively infected cell half-life inferred from these data represents the “functional” half-life rather than a “chronological” half-life. If for example, the average productively infected cell exhibits an exponential increase in viral transcription between days 1 and 3 post-infection, a slight shortening of the chronological lifespan of the cell could have a disproportionately large effect on viral production (burst size). However, such an effect would in fact be perceived in the model used here as a relatively large change in the functional productive lifespan of the infected cell and would be expected to alter first phase plasma RNA kinetics.

The observed one log increase of VL following CD8+ T-cell depletion would require, to first approximation in the timescale studied, a 10-fold increase in productive cell lifespan for the mathematical model used here. Such a change in lifespan is unlikely to be missed even with the study of relatively few animals. To illustrate, we can approximate the most optimistic estimate for the CTL effect on clearance of productively infected cells by determining the difference between the geometric mean of the upper bounds of the 95% confidence intervals for the death rate of the control animals (−0.796 day^−1^) and the geometric mean for the lower bounds of the 95% CI for death rate among the depleted animals (−.30 day^−1^). The mean t1/2 for the depleted animal group of 1.24 day would decrease to 0.66 day if we add the calculated upper limit of the CTL effect of −0.496 day^−1^. However, this approximate two-fold change in t1/2 should only account for a two-fold rise in VL following depletion and not the observed 10-fold change.

A limited role for CTL killing of productively infected cells, although at first surprising, is further supported by several intriguing published observations. For example, first phase plasma HIV RNA decay has been noted to be relatively invariant whether HIV+ patients were treated during early or late stage disease when immune responses would be expected to be robust and waning, respectively [32],[33]. Correspondingly, following structured treatment interruption of HIV suppressive antiretroviral treatment, viral rebound rates do not correlate with the magnitude of HIV specific CD8+ T-cell responses [34]. A final set of observations in accord with the current result is the similarity of viral kinetics of SIV_sm_ following treatment of infected sooty mangabeys and macaques [35] even though in the former, the SIV specific-immune responses tend to be low [36] and CD8 T-cell depletion of sooty mangabeys does not elicit increases in viral load comparable to that seen in macaques [37].

The simultaneous conclusions that CD8+ T-cells exert strong antiviral effects *in vivo* but that the mechanism of viral control is not through the recognition and killing of productively infected cells raises obvious questions about the nature of the CD8+ T-cell antiviral effect(s). In the earlier study by Jin, very frequent plasma sampling immediately after administration of the CD8+ T-cell depleting antibody in two animals allowed the authors to detail the kinetics of increase in SIV VL [7]. The rapid increases in VL were found to be compatible with CD8+ T-cell effects that prevented new infections as might be expected from the elaboration of infection-blocking chemokines or effects that impaired SIV transcription as might be expected with the cell antiviral factor (CAF) [27] but not with CTL effects alone. Because we focused our study on obtaining frequent viral load measurements at week 13 and beyond to accurately define first phase plasma virus decay, we did not have the ability to perform similar immediate post-depletion analyses. Further studies are needed to better characterize and quantify these non-lytic antiviral effects.

The conclusion that CTL activity does not substantially contribute to the clearance of productively infected cells does not imply that CTL activity is irrelevant to viral control. We surveyed viral gene sequences for other markers of CTL effects that might distinguish CTL recognition and killing of infected cells prior to or during viral production. The observation of positive selection pressure acting on *nef* but not *gag* is in alignment with the lack of impact of CD8+ T-cell depletion on first phase plasma virus decay. Because the model used here to describe viral dynamics relates plasma viral decay to the turnover of cells already producing HIV virions, the half-life of productive cells need not change if CTL killing occurred through targeting of Nef (or another early HIV gene product) expressing cells prior to virion production [38],[39]. In contrast, CTL targeting Gag epitopes would be expected to affect measured half-life of productively infected cells to a greater degree. The ability of Nef to downregulate MHC class I provides one possible explanation for why the vulnerability of SIV (and HIV) infected cells to CTL attack is restricted to the early steps of the viral lifecycle (prior to production of threshold levels of Nef needed to downregulate class I and before Gag expression and virion production commence) [40],[41]. Such infected cells expressing Nef could be recognized and cleared by CTL but, because they do not yet contribute to viral production, clearance of these cells would not be reflected in the clearance/death rate (δ) of productively infected cells calculated from first phase plasma virus decay. Instead, the effect of removing these cells would be likened to a reduction in the infection rate. Recent observations that a large proportion of HIV production by infected T-lymphocytes occurs in the subset of CD4− CD8− double negative cells is also compatible with the hypothesis that by the time virion production commences, CD4 and by inference MHC I downregulation has already occurred [42]. Parenthetically, it appears that, in all three cases, the Nef variants that increased in proportion following CD8 T-cell depletion were variants that differed from the consensus sequence of the inoculum (Figure 4). While this suggests that the CTL selective pressure may have been greater against these earlier CTL escape forms than against the inoculum consensus strain, the lack of functional immunologic assays limits our ability to confirm this.

These results and the questions they raise point to a need for more work to be done to better understand the full spectrum of CD8+ T-cell-mediated antiviral effects and the factors that limit CTL capable of targeting the productive stages of the viral life-cycle. As these data argue against CD8+ T-cell-mediated cytolytic activity as the likely mechanism for clearance of productively infected cells, one is left to speculate that viral production by an infected cell is limited by viral cytopathic effects or by as yet unappreciated or unrecognized innate or humoral immune mechanisms that either kill infected cells or drastically down-modulate viral transcription [43],[44]. Refinement of present models of viral and cellular dynamics together with focused research to track the fate of infected cells *in vivo* may provide new insights into these cryptic antiviral mechanisms that could be exploited to treat and prevent HIV disease in the future.

## Supporting Information

Figure S1 Median, plasma SIV RNA (Log copies/ml) over the course of the experiment for all animals. A heavy black arrow marks spontaneous peak VL following primary infection, light arrow indicates start of cM-T807 depletion, hatched horizontal bar shows period of treatment with PMPA FTC. Whiskers show range of values.
(0.09 MB TIF)

Figure S2 Evolution of SIV Gag post depletion of CD8+ cells. Data from 3 fully depleted animals (27562, 32906, 33580) and 2 non-depleted control animals (27988, 28889) are shown. Sequence alignments of SIV Gag sequences (SIVmac251 positions 1–150); sequence names contain animal identity, days post-infection (dp) and arbitrary clone designation. Dashes indicate identity to autologous consensus sequence, asterisks indicate premature stop codons, dots symbolize gaps (deletions), and question marks represent unresolvable sites (due to nucleotide ambiguity). There were no positions in Gag with statistically significant differences in amino acid distribution between day 84 and day 91 (based on a Fisher's Exact Test).
(0.11 MB DOC)

Figure S3 Cumulative behavior of synonymous and nonsynonymous substitutions across gag sequence. A) Data from 3 fully depleted animals. B) Data from 2 non-depleted control animals. Green lines = nonsynonymous mutations, red lines = synonymous substitutions.
(0.79 MB TIF)
