# Supplementary figures and images for: In Vivo CD8+ T-Cell Suppression of SIV Viremia Is Not Mediated by CTL Clearance of Productively Infected Cells
Source: PLoS Pathog. 2010 Jan 29;6(1):e1000748. doi: 10.1371/journal.ppat.1000748 (PMC2813272; doi:10.1371/journal.ppat.1000748)

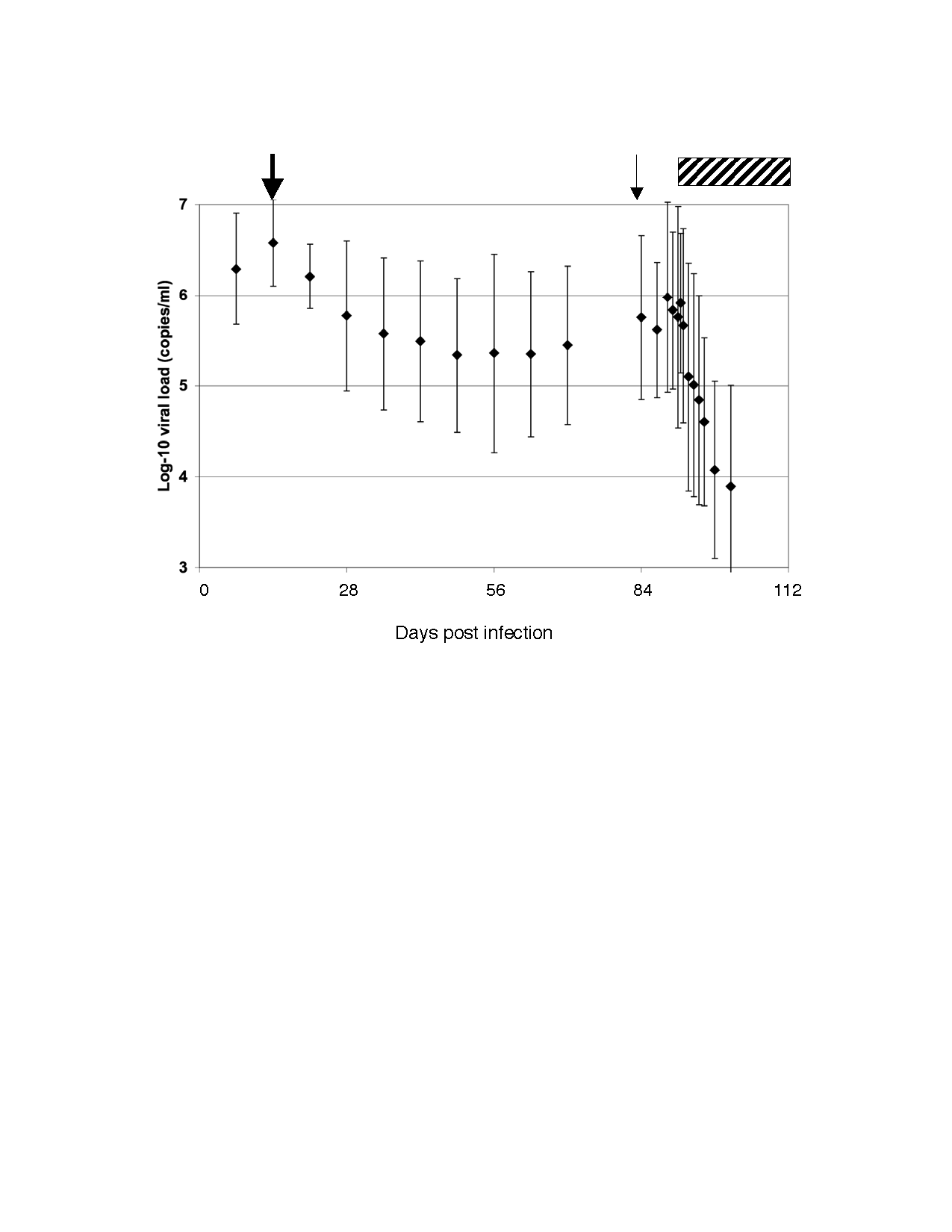

Supplement: Figure S1 — Median, plasma SIV RNA (Log copies/ml) over the course of the experiment for all animals. A heavy black arrow marks spontaneous peak VL following primary infection, light arrow indicates start of cM-T807 depletion, hatched horizontal bar shows period of treatment with PMPA FTC. Whiskers show range of values. (0.09 MB TIF) [file ppat.1000748.s001.tif]

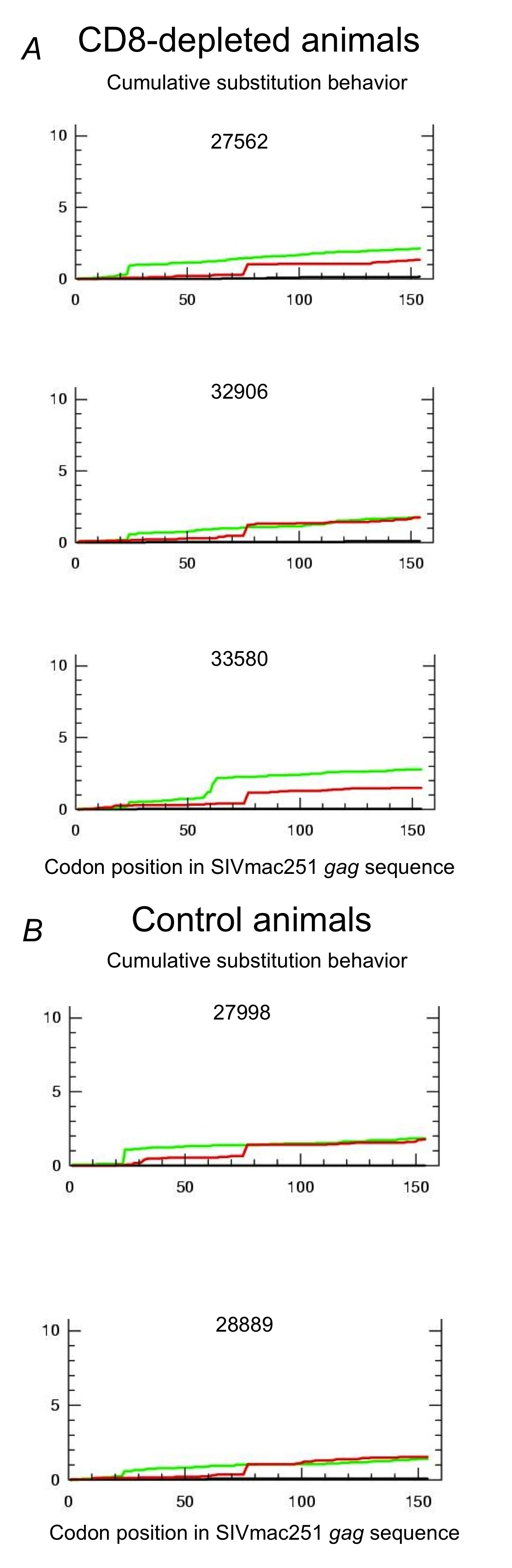

Supplement: Figure S3 — Cumulative behavior of synonymous and nonsynonymous substitutions across gag sequence. A) Data from 3 fully depleted animals. B) Data from 2 non-depleted control animals. Green lines = nonsynonymous mutations, red lines = synonymous substitutions. (0.79 MB TIF) [file ppat.1000748.s003.tif]
