# Supplementary material for: In Vivo CD8+ T-Cell Suppression of SIV Viremia Is Not Mediated by CTL Clearance of Productively Infected Cells
Source: PLoS Pathog. 2010 Jan 29;6(1):e1000748. doi: 10.1371/journal.ppat.1000748 (PMC2813272; doi:10.1371/journal.ppat.1000748)
Supplement: Figure S2 — Evolution of SIV Gag post depletion of CD8+ cells. Data from 3 fully depleted animals (27562, 32906, 33580) and 2 non-depleted control animals (27988, 28889) are shown. Sequence alignments of SIV Gag sequences (SIVmac251 positions 1–150); sequence names contain animal identity, days post-infection (dp) and arbitrary clone designation. Dashes indicate identity to autologous consensus sequence, asterisks indicate premature stop codons, dots symbolize gaps (deletions), and question marks represent unresolvable sites (due to nucleotide ambiguity). There were no positions in Gag with statistically significant differences in amino acid distribution between day 84 and day 91 (based on a Fisher's Exact Test). (0.11 MB DOC) [file ppat.1000748.s002.doc]

CD8-depleted animals

**27562**

Consensus MGARNSVLSG KKADELEKIR LRPNGKKKYM LKHVVWAANE LDRFGLAESL 50

27562_84dp_10 ---------- ---------- ---------- ---------- ----------

27562_84dp_11 ---------- ---------- ---------- ---------- ----------

27562_84dp_12 ---------- ---------- ---G------ ---------- ----------

27562_84dp_13 ---------- ---------- ---------- ---------- ----------

27562_84dp_14 ---------- ---------- ---------- ---------- ----------

27562_84dp_15 ---------- ---------- ---------- ---------- ----------

27562_84dp_17 ---------- ---------- ---------- ---------- ----------

27562_84dp_18 ---------- ---------- ---G------ ---------- ----------

27562_84dp_19 ---------- ---------- ------N--- ---------- ----------

27562_84dp_1 ---------- ---------- ---?------ ---------- ----------

27562_84dp_2 ---------- ---------- ---------- ---------- ----------

27562_84dp_20 ---------- ---------- ---------- ---------- ----------

27562_84dp_21 -------S-- ---------- ---------- ---------- ----------

27562_84dp_22 ---------- ---------- ---------- ---------- ----------

27562_84dp_23 ---------- ---------- ---G------ ---------- ----------

27562_84dp_24 ---------- ---------- ---------- ---------- ----------

27562_84dp_25 ---------- ---------- ---------- ---------- ----------

27562_84dp_26 ---------- -------R-- ---------- ---------- ----------

27562_84dp_27 ---------- ---------- ---------- ---------- --------N-

27562_84dp_28 ---------- ---------G ---------- ---------- ----------

27562_84dp_29 ---------- -*-------- ---------- ---------- ----------

27562_84dp_3 ---------- ---------- ---G------ ---------- ----------

27562_84dp_30 ---------- ---------- ---------- ---------- ----------

27562_84dp_4 ---------- ---------- ---------- ---------- ----------

27562_84dp_5 ---------- ---------- ---------- ---A------ ----------

27562_84dp_6 ---------- ---------- ---G------ ---------- ----------

27562_84dp_7 ---------- ---------- ---------- ---------- ----------

27562_84dp_8 ---------- ---------- ---------- ---------- ----------

27562_84dp_9 ---------- ---------- ---G------ ---------- ----------

27562_91dp_10 ---------- ---------- ---G------ ---------- ----------

27562_91dp_11 ---------- ---------- ---------- ---------- -V--------

27562_91dp_12 ---------- ---------- ---G------ ---------- ----------

27562_91dp_13 ---------- ---------- ----E----- ---------- ----------

27562_91dp_15 ---------- ---------- ---------- ---------- ----------

27562_91dp_16 ---------- ---------- ---------- ---------- ----------

27562_91dp_17 ---------- --------F- ---------- ---------- ----------

27562_91dp_18 ---------- ---------- ---------- ---------- ----------

27562_91dp_19 ---------- ---------- ---------- ---------- ----------

27562_91dp_1 ---------- ---------- ---------- ---------- ---------P

27562_91dp_20 ---------- ---------- ---------- ---------- ----------

27562_91dp_21 ---------- ---------- ---------- ---------- ----------

27562_91dp_23 ---------- ---------- ---------- ---------- ----------

27562_91dp_24 ---------- ---------- ---------- ---------- ----------

27562_91dp_27 ---------- ---------- ---------- ---------- ----------

27562_91dp_28 ---------- ---------- ---------- ---------- ----------

27562_91dp_2 --T------- ---------- ---------- ---------- ---------P

27562_91dp_3 ---------- ---------- ---------- ---------- ----------

27562_91dp_4 ---------- ---------- ---G------ ---------- ----------

27562_91dp_5 ---------- ----V----- ---------- ---------- --*-------

27562_91dp_8 ---------- ---------- --L------- ---------- ----------

Consensus LENKEGCQKI LSVLAPLVPT GSENLKSLYN TVCVIWCIHA EEKVKHTEEA 100

27562_84dp_10 ---------- ---------- ---------- ---------- ----------

27562_84dp_11 ---------- ---------- ---------- ---------- ----------

27562_84dp_12 ---------- ---------- ---------- ---------- ----------

27562_84dp_13 ---------- ---------- ---------- ---------- ----------

27562_84dp_14 ---------- ---------- ---------- ---------- --------G-

27562_84dp_15 ---------- -------M-- ---------- ---------- ----------

27562_84dp_17 ---------- ---------- ---------- ---------- ----------

27562_84dp_18 ---------- ---------- ---------- ---------- ----------

27562_84dp_19 ---------- ---------- ---------- ---------- ----------

27562_84dp_1 ---------- ---------- ---------- ---------- ----------

27562_84dp_2 ---------- ---------- ---------- ---------- ----------

27562_84dp_20 ---------- ---------- ---------- ---------- ----------

27562_84dp_21 ---------- ---------- ---------- -------V-- ----------

27562_84dp_22 ---------- ---------- ---------- ---------- ----------

27562_84dp_23 ---------- ---------- ---------S ---------- ----------

27562_84dp_24 ---------- ---------- ---------- ---------- ----------

27562_84dp_25 ---------- ---------- ---------- ---------- ----------

27562_84dp_26 ---------- ---------- ---------- ---------- ----------

27562_84dp_27 ---------- ---------- ---------- ---------- ----------

27562_84dp_28 ---------- ---------- ---------- ---------- ----------

27562_84dp_29 ---------- ---------- ---------- ---------- ----------

27562_84dp_3 ---------- ---------- ---------- ---------- ----------

27562_84dp_30 ---------- ---------- ---------- ---------- ----------

27562_84dp_4 ---------- ---------- ---------- ---------- ----------

27562_84dp_5 ---------- ---------- ---------- ---------- ----------

27562_84dp_6 ---------- ---------- ---------- ---------- ----------

27562_84dp_7 ---------- ---------- ---------- ---------- ----------

27562_84dp_8 ---------- ---------- ---------- ---------- ----------

27562_84dp_9 ---------- ---------- ---------- ---------- ----------

27562_91dp_10 --------R- ---------- ---------- ---------- ----------

27562_91dp_11 ---------- ---------- ---------- ---------- ----------

27562_91dp_12 ---------- ---------- ---------- ---------- ----------

27562_91dp_13 ---------- ---------- ---------- ---------- ----------

27562_91dp_15 ------*--- ---------- ---------- ---------- ----------

27562_91dp_16 ---------- ---------- ---------- ---------- ----------

27562_91dp_17 ---------- ---------- ---------- ---------- ----------

27562_91dp_18 ---------- ---------- ---------- ---------- ----------

27562_91dp_19 ---------- -------M-- -----E---- ---------- ----------

27562_91dp_1 ---?------ -----?---- ---------- ---------- ----------

27562_91dp_20 ---------- ---------- ---------- ---------- ----------

27562_91dp_21 ---------- ---------- ---------- ---------- ----------

27562_91dp_23 ---------- ---------- ---------- ---------- ----------

27562_91dp_24 ---------- ---S------ ---------- ---------- ----------

27562_91dp_27 ---------- ---------I ---------- ---A------ ----------

27562_91dp_28 ---------- ---------- ---------- ---------- ----------

27562_91dp_2 ---?------ ---------- ---------- ---------- ----------

27562_91dp_3 ---------- ---------- ---------- ---------- ----------

27562_91dp_4 ---------- ---------- ---------- ---------- ----N-----

27562_91dp_5 ---------- ---------- ---------- ---------- ----------

27562_91dp_8 ---------- ---------- --G------- -----?---- ----------

Consensus KQIVQRHLVV ETGTAETMPK TSRPTAPSSG RGGNYPVQQI GGNYVHLPLS 150

27562_84dp_10 ---------- ---------- ---------- ---------- ----------

27562_84dp_11 ---------- ---------- ---------- ---------- ----------

27562_84dp_12 ---------- ---------- ---------- ---------- ----------

27562_84dp_13 ---------- ---------- ---------- ---------- ----------

27562_84dp_14 ---------- -A-------- ---------- ---------- ----------

27562_84dp_15 ---------- ---------- ---------- ---------- ----------

27562_84dp_17 ---------- ---------- ---------- ---------- ----------

27562_84dp_18 ---------- ---------- ---------- ---------- ----------

27562_84dp_19 ---------- ---------- ---------- ---------- ----------

27562_84dp_1 ---------- ---------- ---------- ---------- ----------

27562_84dp_2 ---------- ---------- ---------- ---------- ----------

27562_84dp_20 ---------- ---------- ---------- ---------- ----------

27562_84dp_21 ---------- ---------- -------P-- ---------- ----------

27562_84dp_22 ---------- ---------- ---------- ---------- ----------

27562_84dp_23 ---------- ---------- ---------- ---------- ----------

27562_84dp_24 ---------- ---------- ---------- ---------- ----------

27562_84dp_25 ---------- ---------- ---------- ---------- ----------

27562_84dp_26 ---------- ---------- ---------- ---------- ----------

27562_84dp_27 ---------- ---------- ---------- ---------- ----------

27562_84dp_28 ---------- ---------- ---------- ---------- ----------

27562_84dp_29 ---------- -A-------- ---------- ---------- ----------

27562_84dp_3 E--------- ---------- ---------- ---------- ----------

27562_84dp_30 ---------- ------A--- ---------- ---------- ----------

27562_84dp_4 ----L----- ---------- ---------- ---------- ----------

27562_84dp_5 ---------- ---------- ---------- ---------- ----------

27562_84dp_6 ---------- ---------- ---------- ---------- ----------

27562_84dp_7 ---------- ---------- ---------- S--------- ----------

27562_84dp_8 ---------- ---------- ---------- ---------- ----------

27562_84dp_9 ---------- ---------- ---------- ---------- --S-------

27562_91dp_10 ---------- ---------- ---------- ---------- ----------

27562_91dp_11 ---------- ---------- ---------- ---------- ----------

27562_91dp_12 ---------- ---------- ---------- ---------- ----------

27562_91dp_13 ---------- ---------- ---------- ---------- ----------

27562_91dp_15 ---------- ---------- ---------- ---------- ----------

27562_91dp_16 ---------- ---------- ---------- ---------- ----------

27562_91dp_17 ---------- ---------- ---------- ---------- ----------

27562_91dp_18 ---------- ---------- ---------- ---------- ----------

27562_91dp_19 ---------- ---------- ---------- ---------- ----------

27562_91dp_1 ---------- ---------- ---------- ---------- ----------

27562_91dp_20 ?--------- ---------- ---------- ---------- ----------

27562_91dp_21 ---------- -------I-- ---------- ---------- ----------

27562_91dp_23 ---------- ---------- ---------- ---------- ----------

27562_91dp_24 ---------- ---------- ---------- ------I--- ----------

27562_91dp_27 ---------- ---------- ---------- ---------- ----------

27562_91dp_28 ---------- ---------- ---------- ---------- ----------

27562_91dp_2 -----G---- ---------- ---------- ---------- ---------G

27562_91dp_3 ---------- ---------- ---------- ---------- ----------

27562_91dp_4 ---------- ---------- ---------- ---------- ----------

27562_91dp_5 ---------- ---------- ---------- ---------- ----------

27562_91dp_8 ---------- ---------- ---------- ---------- ----------

**32906**

Consensus MGARNSVLSG KKADELEKIR LRPNGKKKYM LKHVVWAANE LDRFGLAESL 50

32906_84dp_1 ---------- ---------- ---------- ---------- ----------

32906_84dp_10 ---------- ---------- ---------- ---------- ----------

32906_84dp_11 ---------- ---------- ---------- ---------- ----------

32906_84dp_13 ---------- ---------- ---------- ---------- ----------

32906_84dp_16 ---------- ---------- ---------- ---------- ----------

32906_84dp_17 ---------- ---------- ---G------ ---------- ----------

32906_84dp_18 ---------- ---------- ---G------ ---------- ----------

32906_84dp_19 ---------- ---------- ---------- ---------- ----------

32906_84dp_2 ---------- ---------- ---G------ ---------- ----------

32906_84dp_21 ---------- ---------- ---------- ---------- ----------

32906_84dp_23 ---------- ---------- ---------- ---------- ----------

32906_84dp_24 ---------- ---------- ---------- ---------- ----------

32906_84dp_25 ---------- ---------- -------R-- ---------- ----------

32906_84dp_26 ---------- ---------- ---------- -?-------- ----------

32906_84dp_27 ---------- ---------- ---------- ---------- ----------

32906_84dp_28 ---------- ---------- ---------- ----I----- ----------

32906_84dp_29 ---------- ---------- ---------- ---------- ----E-----

32906_84dp_3 ---------- ---------- ---------- ---------- ----------

32906_84dp_4 ---------- ---------- ---------- ---------- ----------

32906_84dp_5 ---------- ---------- ---------- ---------- ----------

32906_84dp_7 ---------- ---------- ---------- ---------- ----------

32906_84dp_8 -R-------- ---------- ---------- ---------- ----------

32906_84dp_9 ---------- ---------- -------R-- ---------- ----------

32906_91dp_1 ---------- ---------- ---------- ---------- ----------

32906_91dp_10 ---------- ---------- ---------- ---------- ----------

32906_91dp_11 ---------- ---------- ---------- ---------- ----------

32906_91dp_12 ---------- -R-------- ---------- ---------- ----------

32906_91dp_13 ---------- ---------- ---------- ---------- ----------

32906_91dp_14 ---------- ---------- ---------- ---------- ----------

32906_91dp_15 ---------- ---------- ---------- ---------- ----------

32906_91dp_16 ---------- ---------- ---------- ---------- ----------

32906_91dp_17 ---------- ---------- ---------- ---------- ----------

32906_91dp_18 ---------- ---------- ---------- ---------- ----------

32906_91dp_20 ---------- ---------- ---------- ---------- ----------

32906_91dp_21 ---------- ---------- ---------- ---------- ----------

32906_91dp_22 ---------- ---------- ---------- ---------- ----------

32906_91dp_23 ---------- ---------- ---G------ ---------- ----------

32906_91dp_24 ---------- ---------- ---------- ---------- ----------

32906_91dp_29 ---------- --------F- ---------- ---------- ----------

32906_91dp_30 ---------- ---------- ---------- ---------- ----------

32906_91dp_4 ---------- ---------- ---G------ ---------- ----------

32906_91dp_5 ---------- ---------- ---------- ---------- ----------

32906_91dp_6 ---------- ---------- ---------- ---------- ----------

32906_91dp_7 ---------- ---------- ---------- ---------- ----------

32906_91dp_8 ----S----- ---------- ---------- ---------- ----------

32906_91dp_9 ---------- ---------- ---------- ---------- ----------

Consensus LENKEGCQKI LSVLAPLVPT GSENLKSLYN TVCVIWCIHA EEKVKHTEEA 100

32906_84dp_1 ---------- ---------- ---------- ---------- ----------

32906_84dp_10 ---------- ---------- ---------- ---------- ----------

32906_84dp_11 ---------- ---------- ---------- ---------- ----------

32906_84dp_13 ---------- ---------- ---------- ---------- ----------

32906_84dp_16 ---------- ---------- ------G--- ---------- ----------

32906_84dp_17 ---------- ---------- ---------- ---------- ----------

32906_84dp_18 ---T------ ---------- ---------- ---------- ----------

32906_84dp_19 ---------- ---------- ---------- ---------- ----------

32906_84dp_2 ---------- ---------- ---------- ---------- ----------

32906_84dp_21 S--------- ---------- ---------- ---------- ----------

32906_84dp_23 ---------- ---------- ---------- ---------- ----------

32906_84dp_24 ---------- ---------- ---------- ---------- ----------

32906_84dp_25 ---------- ---------- ---------- ---------- G---------

32906_84dp_26 ---------- ---------- ---------- ---------- ----------

32906_84dp_27 ---------- ---------- ---------- ---------- ----------

32906_84dp_28 ---------- ---------- ---------- ---------- ----------

32906_84dp_29 ---------- ---------- ---------- ---------- ----------

32906_84dp_3 ---------- ---------- ---------- ---------- ----------

32906_84dp_4 ---------- ---------- ---------- ---------- ----------

32906_84dp_5 ---------- ---------- ---------- ---------- ----------

32906_84dp_7 ---------- ---------- ---------- ---------- ----------

32906_84dp_8 ---------- ---------- ---------- ---------- ----------

32906_84dp_9 ---------- ---------- ---------- ---------- ----------

32906_91dp_1 ---------- ---------- ---------- ---------- ----------

32906_91dp_10 ---------- ---------- ---------- ---------- ----------

32906_91dp_11 ---------- ---------- ---------- ---------- ----------

32906_91dp_12 ---------- ---------- ---------- ---------- ----------

32906_91dp_13 ---------- ---------- ---------- ---------- ----------

32906_91dp_14 ---------- ---------- ---------- ---------- ----------

32906_91dp_15 ------R--- ---------- ---------- ---------- ----------

32906_91dp_16 ---------- ----D----- ---------- ---------- ----------

32906_91dp_17 ---T------ ---------- ---------- ---------- ----------

32906_91dp_18 ---------- ---------- ---D------ ---------- ----------

32906_91dp_20 ---------- ---------- ---------- ---------- ----------

32906_91dp_21 ---------- ---------- ---------- ---------- ----------

32906_91dp_22 ---------- -L-------- ---------- ---------- ----------

32906_91dp_23 ---------- ---------- ---------- ---------- ----------

32906_91dp_24 ---------- ---------- ---------- ---------- ----------

32906_91dp_29 ---------- ---------- ---------- ---------- ----------

32906_91dp_30 ---------- ---------- ---------- ---------- ----------

32906_91dp_4 ---------- ---------- ---------- ---------- ----------

32906_91dp_5 ---------- ---------- ---------- ---------- ----------

32906_91dp_6 ---------- ---------- ---------- ---------- ----------

32906_91dp_7 ---------- ---------- ---------- ---------- ----------

32906_91dp_8 ---------- ---------- ---------- ---------- ----------

32906_91dp_9 ---------- ---------- ---------- ---------- ----------

Consensus KQIVQRHLVV ETGTAETMPK TSRPTAPSSG RGGNYPVQQI GGNYVHLPLS 150

32906_84dp_1 -K-------- ---------- ?--------- ---------- ----------

32906_84dp_10 --M------- ---------- ---------- ---------- ----------

32906_84dp_11 ---------- ---------- ---------- ---------- ----------

32906_84dp_13 ---------- ---------- ---------- ---------- ----------

32906_84dp_16 ---------- ---------- ---------- ---------- ----------

32906_84dp_17 ---------- ---------- ---------- ---------- ----------

32906_84dp_18 ---------- ---------- ---------- ---------- ----------

32906_84dp_19 ---------- --E------- ---------- ---------- ----------

32906_84dp_2 ---------- ---------- ---------- ---------- ----------

32906_84dp_21 ---------- ---------- ---------- ---------- ----------

32906_84dp_23 ---------- ---------- ---------- ---------- ----------

32906_84dp_24 ---------- ---------- ---------- ---------- ----------

32906_84dp_25 ---------- -----V---- ---------- ---------- ----------

32906_84dp_26 ---------- ---------- ---------- ---------- ----------

32906_84dp_27 ---------- ---------- ---------- ---------- ----------

32906_84dp_28 ---------- ---------- ---------- ---------- ----------

32906_84dp_29 ---------- ------A--- ---------- ---------- ----------

32906_84dp_3 ---------- ---------- ---------- ---------- ----------

32906_84dp_4 -----K---- ---------- -------A-D ---------- ----------

32906_84dp_5 ---------- ---------- ---------- ---------- ----------

32906_84dp_7 ---------- ---------- ---------- ---------- ----------

32906_84dp_8 ---------- ---------- ---------- ---------- ----------

32906_84dp_9 ---------- ---------- ---------- ---------- ----------

32906_91dp_1 ---------- ---------- ---------- ---------- ----------

32906_91dp_10 ---------- ---------- ---------- ---------- ----------

32906_91dp_11 ---------- ---------- ---------- ---------- ----------

32906_91dp_12 ---------- ---------- ---------- ---------- ----------

32906_91dp_13 ---------- ---------- ---------- ---------- ----------

32906_91dp_14 ---------- ---------- ---------- ---------- ----------

32906_91dp_15 ---------- ---------- ---------- ---------- ----------

32906_91dp_16 ---------- ---------- ---------- ---------M ----------

32906_91dp_17 ---------- ----T----- ---------- ---------- ----------

32906_91dp_18 ---------- ---------- ---------- ---------- ----------

32906_91dp_20 ---------- ---------- ---------- ---------- ----------

32906_91dp_21 ---------- ---------- ---------- ---------- ----------

32906_91dp_22 ---------- ---------- ---------- ---------- --------S-

32906_91dp_23 ---------- ------A--- ---------- ---------- ----------

32906_91dp_24 ---------- ---------- ---------- ---------- ----------

32906_91dp_29 ---------- ---------- ---------- ---------- ----------

32906_91dp_30 ---------- ---------- ---------- ---------- ----------

32906_91dp_4 ---------- ---------- ---------- ---------- ----------

32906_91dp_5 ---------- ---------- ---------- ---------- ----------

32906_91dp_6 ---------- -A-------- --*------- ---------- ----------

32906_91dp_7 ---------- ---------- ---------- ---------- ----------

32906_91dp_8 ---------- ---------- ---------- ---------- ----------

32906_91dp_9 ---------- ---------- ---------- ---------- ----------

**33580**

Consensus MGARNSVLSG KKADELEKIR LRPGGKKKYM LKHVVWAANE LDRFGLAESL 50

33580_84dp_1 ---------- ---------- ---------- ---------- ----------

33580_84dp_10 ---------- ---------- ---N------ ---------- ----------

33580_84dp_11 ---------- ---------- ---N------ ---------- ----------

33580_84dp_12 ---------- ---------- ---------- ----I----- ----------

33580_84dp_13 ---------- ---------- ---------- ---------- ----------

33580_84dp_15 ---------- ---------- ---------- ---------- ----------

33580_84dp_16 ---------- ---------- ---------- ---------- ----------

33580_84dp_17 ---------- ---------- ---------- ---------- ----------

33580_84dp_18 ---------- ---------- ---------- ---------- ----------

33580_84dp_19 ---------- ---------- ---------- ---------- ----------

33580_84dp_2 ---------- ---------- ---------- ---------- ----------

33580_84dp_20 ---------- ---------- ---------- ---------- ----------

33580_84dp_21 ---------- ---------- ---------- ---------- ----------

33580_84dp_22 --T------- ---------- ---------- ---------- ----------

33580_84dp_23 -------*-- ---------- ---------- ---------- ----------

33580_84dp_24 ---------- ---------- ---------- ---------- ----------

33580_84dp_25 ---------- ---------- ---------- ---------- ----------

33580_84dp_26 ---------- ---------- ---------- ---------- --*-------

33580_84dp_27 ---------- ---------- ---------- ---------- ---L------

33580_84dp_28 ---------- ---------- ---------- ---------- ----------

33580_84dp_29 ---------- ---------- ---------- ---------- ----------

33580_84dp_3 ---------- ---------- ---------- ---------- ----------

33580_84dp_30 ---------- ---------- ---------- ---------- ----------

33580_84dp_4 ---------- ---------- ---------- ---------- ----------

33580_84dp_5 ---------- ---------- ---------- --------D- ----------

33580_84dp_6 ---------- ---------- ---------- ---------- -----*----

33580_84dp_7 ---------- ---------- ---------- ---------- ----------

33580_84dp_8 ---------- ---------- ---------- ---------- ----------

33580_84dp_9 ---------- ---------- ---------- ---------- ----------

33580_91dp_1 ---------- ---------- ---------- ---------- ----------

33580_91dp_10 ---------- ---------- ---------- ---------- ----------

33580_91dp_11 ---------- ---------- ---------- ---------- ----------

33580_91dp_12 ---------- --------T- ---------- ---------- ----------

33580_91dp_13 ---------- ---------- ---------- ---------- ----------

33580_91dp_14 ---------- ---------- ---------- ---------- ----------

33580_91dp_15 ---------- ---------- ---------- ---------- ----------

33580_91dp_16 ---------- ---------- ---------- ---------- ----------

33580_91dp_17 ---------- ---------- ---------- ---------- ----------

33580_91dp_18 ---------- ---------- -------R-- ---------- ----------

33580_91dp_19 ---------- ---------- ---------- ---------- ----------

33580_91dp_20 ---------- ---------- Q--------- ---------- ----------

33580_91dp_21 ---------- ---------- -------?-- ---------- ----------

33580_91dp_23 ---------- ---------- ---N------ ---------- ----------

33580_91dp_24 ---------- ---------- ---S------ ---------- ----------

33580_91dp_25 ---------- ---------- ---------- ---------- ----------

33580_91dp_26 ---------- ---------- ---------- ---------- ----------

33580_91dp_27 ---------- ---------- ---------- ---------- ----------

33580_91dp_28 ---------- ---------- ---------- ---------- ----------

33580_91dp_29 ---------- ---------- ---------- ---------- ----------

33580_91dp_3 ---------- ---------- ---------- ---------- ----------

33580_91dp_30 ---------- ---------- ---N------ ---------- ----------

33580_91dp_5 ---------- ---------- ---------- ---------- ----------

33580_91dp_8 ---------- ---------- --S------- ---------- ----------

33580_91dp_9 ---------- ---------- ---------- ---------- ----------

Consensus LENKEGCQKI ISVLAPLVPT GSENLKSLYN TVCVIWCIHA EEKVKHTEEA 100

33580_84dp_1 ---------- -------A-- ---------- ---------- ----------

33580_84dp_10 ---R----R- L--------- ---------- ---------- ----------

33580_84dp_11 ---------- ---------- ---------- A--------- ----------

33580_84dp_12 ---------- L-I------- ---------- ---------- ----------

33580_84dp_13 ---------- ---------- ---------- ---------- ----------

33580_84dp_15 ---------- ---------- ---------- ---------- ----------

33580_84dp_16 -------K-- L--------- ---------- ---------- ----------

33580_84dp_17 ---------- ---------- ---------- ---------- ----------

33580_84dp_18 ---------- ---------- ---------- ---------- ----------

33580_84dp_19 --Y------- ---------- ---------- ---------- ----------

33580_84dp_2 ---------- L-I------- ---------- ---------- ----------

33580_84dp_20 ---------- ---------- ---------- ---------- ----------

33580_84dp_21 ---------- ---------- ---------- ---------- ----------

33580_84dp_22 ---------- LL-------- ---------- ---------- ----------

33580_84dp_23 ---------- ---------- ---------- ---------- ----------

33580_84dp_24 ---------- LL-------- ---------- ---------- ----------

33580_84dp_25 ---------- LL-------- ---------- ---------- ----------

33580_84dp_26 ---------- ---------- ---------- ---------- ----------

33580_84dp_27 --------R- L--------- ---------- ---------- ----------

33580_84dp_28 ---------- ---------- ---------- ---------- ----------

33580_84dp_29 -------R-- L--------- ---------- ---------- ----------

33580_84dp_3 ---------- ---------- ---------- ---------- ----------

33580_84dp_30 -------R-- L--------- ---------- ---------- ----------

33580_84dp_4 ---------- L-I------- ---------- ---------- ----------

33580_84dp_5 ---------- LL-------- ---------- ---------- ----*-----

33580_84dp_6 ---------- ---------- ---------- ---------- ----------

33580_84dp_7 --------R- L--------- ---------- ---------- ----------

33580_84dp_8 ---------- ---------- D--------- ---------- ----------

33580_84dp_9 ------R--- ---------- ---------- ---------- ----------

33580_91dp_1 ---------- ---------- ---------- ---------- ----------

33580_91dp_10 ---------- ---------- ---------- ---------- ----------

33580_91dp_11 ---------- L-I------- ---------- ---------- ----------

33580_91dp_12 ---------- ---------- ---------- ---------- ----------

33580_91dp_13 ---------- ---------- ---------- ---------- ----------

33580_91dp_14 --------R- L--------- ---------- ---------- ----------

33580_91dp_15 ---------- ---------- ---------- ---------- ----------

33580_91dp_16 ---------- ---------- ---------- ---------- ----------

33580_91dp_17 ---------- LL-------- ---------- ---------- ----------

33580_91dp_18 ---------- ---------- ---------- ---------- ----------

33580_91dp_19 ---------- ---------- ---------- ---------- ----------

33580_91dp_20 ---------- L-I------- ---------- ---------- ----------

33580_91dp_21 ---------- LL-------- ---------- -----R---- ----------

33580_91dp_23 ---------- ---------- ---------- -----*---- ----------

33580_91dp_24 -------R-- L--------- ---------- ---------- ----------

33580_91dp_25 ---------- L-I------- ---------- ---------- ----------

33580_91dp_26 ---------- L-I------- ---------- ---------- ----------

33580_91dp_27 --------R- L--------- ---------- ---------- ----------

33580_91dp_28 ---------- ---------- ---------- ---------- ----------

33580_91dp_29 -------R-- L--------- ---------- ---------- ----------

33580_91dp_3 -------R-- L--------- ---------- ---------- ----------

33580_91dp_30 ---------- L--------- ---------- ---------- ----------

33580_91dp_5 ---------- ---------- ---------- ---------- ----------

33580_91dp_8 ---------- T--------- ---------- ---------- ----------

33580_91dp_9 ---------- ---------- ---------- ---------- ----------

Consensus KQIVQRHLVV ETGTAETMPK TSRPTAPSSG RGGNYPVQQI GGNYVHLPLS 150

33580_84dp_1 ---------- ---------- ---------- ---------- ----------

33580_84dp_10 ---------- -A-------- ---------- ---------- ----------

33580_84dp_11 ---------- ---------- ---------- ---------- ----------

33580_84dp_12 ---------- ---------- ---------- ---------- ----------

33580_84dp_13 ---------- ---------- ---------- ---------- ----------

33580_84dp_15 ----*----- ---------- ---------- ---------- ----------

33580_84dp_16 ---------- ---------- ---------- ---------- ----------

33580_84dp_17 ---------- ---------- ---------- ---------- ----------

33580_84dp_18 ---------- ---------- ---------- ---------- ----------

33580_84dp_19 ---------- ---------- ---------- ---------- ----------

33580_84dp_2 ---------- ---------- ---------- ---------- ----------

33580_84dp_20 ---------- ---------- ---------- ---------- ----------

33580_84dp_21 ---------- ---------- ---------- ---------- ----------

33580_84dp_22 ---------- ---------- ---------- ---------- ----------

33580_84dp_23 ---------- ---------- ---------- ---------- ----------

33580_84dp_24 ---------- ---------- ---------- ---------- ----------

33580_84dp_25 ---------- ---------- ---------- ---------- ----------

33580_84dp_26 ---------- ---------- ---------- ---------- ----------

33580_84dp_27 ---------- ---------- ---------- ---------- ----------

33580_84dp_28 ---------- -------T-- ---------- ---------- ----------

33580_84dp_29 ---------- ---------- ---------- ---------- ----------

33580_84dp_3 ---------- ---------- ---------- ---------- ----------

33580_84dp_30 ---------- ---------- ---------- ---------- ----------

33580_84dp_4 ---------- ---------- ---------- ---------- ----------

33580_84dp_5 ---------- ---------- ---------- ---------- ----------

33580_84dp_6 ---------- ---------- ---------- ---------- ----------

33580_84dp_7 ---------- ---------- ---------- K--------- ----------

33580_84dp_8 ---------- K--------- ---------- ---------- ----------

33580_84dp_9 ---------A ---------- ---------- ---------- ----------

33580_91dp_1 ---------- ---------- ---------- --------R- ----------

33580_91dp_10 ---------- ---------- ---------- ---------- ----------

33580_91dp_11 ---------- ---------- ---------- ---------- ----------

33580_91dp_12 ---------- ---------- ---------- ---------- ----------

33580_91dp_13 ---------- ---------- ---------- ---------- ----------

33580_91dp_14 ---------- ---------- ---------- ---------- ----------

33580_91dp_15 ---------- ---------- ---------- ---------- ----------

33580_91dp_16 ---------- ---------- ---------- ---------- ----------

33580_91dp_17 ---------- ---------- ---------- ---------- ----------

33580_91dp_18 ---------- ---------- ---------- ---------- ----------

33580_91dp_19 ---------- ---------- ---------- ---------- ----------

33580_91dp_20 ---------- ---------- ---------- ---------- ----------

33580_91dp_21 R--------- ---------- ---------- ---------- -S--------

33580_91dp_23 ---------- ---------- ---------- ---------- ----------

33580_91dp_24 ---------- ---------- ---------- ---------- ----------

33580_91dp_25 ---------- ---------- ---------- ---------- ----------

33580_91dp_26 ---------- ---------- ---------- ---------- ----------

33580_91dp_27 ---------- ---------- ---------- ---------- ----------

33580_91dp_28 ---------- ---------- ---------- ---------- ----------

33580_91dp_29 ---------- ---------- ---------- ---------- ----------

33580_91dp_3 ---------- ---------- ---------- ---------- ----------

33580_91dp_30 ---------- ---------- ---------- ---------- ----------

33580_91dp_5 ---------- ---------- ---------- ---------- ----------

33580_91dp_8 ---------- ---------- ---------- ---------- ------P---

33580_91dp_9 ---------- ---------- ---------- ---------- ----------

Control animals

**27988**

Consensus MGARNSVLSG KKADELEKIR LRPNGKKKYM LKHVVWAANE LDRFGLAESL 50

27988_wk.12_1 ---------- ---------- ---------- ---------- ---------P

27988_wk.12_10 ---------- ---------- ---G------ ---------- ----------

27988_wk.12_12 ---------- ---------- ---------- ---------- ----------

27988_wk.12_13 ---------- ---------- ---------- ---------- ----------

27988_wk.12_14 ---------- ---------- ---------- ---------- ----------

27988_wk.12_15 ---------- ---------- ---------- -----R---- ----------

27988_wk.12_16 ---------- ---------- ---G------ ---------- ----------

27988_wk.12_17 -D-------- ---------- ---G------ ---------- ----------

27988_wk.12_18 ---------- ---------- ---------- ---------- ----------

27988_wk.12_2 ---------- ---------- ---------- ---------- ----------

27988_wk.12_20 ---------- ---------- ---------- ---------- ----------

27988_wk.12_21 ---------- ---------- ---------- ---------- ----------

27988_wk.12_22 ---------- ---------- ---------- ---------- ----------

27988_wk.12_23 ---------- ---------- ---------- ---------- ----------

27988_wk.12_25 ---------- ---------- ---G------ ---------- ----------

27988_wk.12_26 ---------- ---------- ---G------ ---------- ----------

27988_wk.12_27 ---------- ---------- ---------- ---------- ----------

27988_wk.12_28 ---------- ---------- ---G------ ---------- ----------

27988_wk.12_29 ---------- ---------- ---G------ ---------- ----------

27988_wk.12_3 ---------- ---------- ---------- ---------- ----------

27988_wk.12_30 ---------- ---------- ---G------ ---------- ----------

27988_wk.12_4 I--------- ---------- ---G------ ---------- ----------

27988_wk.12_6 ---------- ---------- ---G------ ---------- ----------

27988_wk.12_7 ---------- ---------- ---G------ ---------- ----------

27988_wk.12_8 ---------- ---------- ---------- ---------- ----------

27988_wk.12_9 ---------- ---------- ---G------ ---------- ----------

27988_wk.13_1 ---------- ---------- ---G------ ---------- ----------

27988_wk.13_10 ---------- -E-------- -------R-- ---------- -----I----

27988_wk.13_11 ---------- ---------- ---------- ---------- ----------

27988_wk.13_12 ---------- ---------- ---------- ---------- ----------

27988_wk.13_13 ---------- ---------- ---G------ ---------- ----------

27988_wk.13_14 ---------- ---------- ---G------ ---------- ----------

27988_wk.13_15 ---------- ---------- ---------- ---------- ----------

27988_wk.13_16 ---------- ---------- ---------- ---------- ----------

27988_wk.13_17 ---------- ---------- ---D------ ---------- ----------

27988_wk.13_18 ---------- ---------- ---------- ---------- ----------

27988_wk.13_19 ---------- ---------- ---------- ---------- ----------

27988_wk.13_2 ---------- ---------- ---G------ ---------- ----------

27988_wk.13_20 ---------- ---------- ---------- ---------- ----------

27988_wk.13_21 ---------- ---------- ---G------ ---------- ----------

27988_wk.13_22 ---------- ---------- ---------- ---------- ----------

27988_wk.13_23 ---------- ---------- ---G------ ---------- ----------

27988_wk.13_24 ---------- ---------- ---------- ---------- ----------

27988_wk.13_25 ---------- ---------- ---G------ ---------- ----------

27988_wk.13_26 ---------- ---------- ---------- ---------- ----------

27988_wk.13_27 ---------- ---------- ---------- M--------- ----------

27988_wk.13_28 ---------- ---------- ---------- ---------- ----------

27988_wk.13_29 ---------- ---------- ---------- ---------- ----------

27988_wk.13_30 ---------- ---------- ---G------ ---------- ----------

27988_wk.13_3 ---------- ---------- ---G------ ---A------ ----------

27988_wk.13_5 ---------- ---------- ---G------ ---------- ----------

27988_wk.13_6 ---------- ---------- ---G------ ---------- ----------

27988_wk.13_7 ---------- ---------- ---G------ ---------- ----------

27988_wk.13_8 ---------- ---------- ---?------ ---------- ----------

Consensus LENKEGCQKI LSVLAPLVPT GSENLKSLYN TVCVIWCIHA EEKVKHTEEA 100

27988_wk.12_1 ---------- ---------- ---------- ---------- ----------

27988_wk.12_10 ---------- ---------- ---------- ---------- ----------

27988_wk.12_12 ---------- ---------- ---------- ---------- ----------

27988_wk.12_13 ---------- ---------- ---------- --R------- ----------

27988_wk.12_14 ---------- ---------- ---------- ---------- ----------

27988_wk.12_15 ---------- ---------- ---------- ---------- ----------

27988_wk.12_16 ---------- ---------- ---------- ---------- ----------

27988_wk.12_17 ---------- ---------- ---------- ---------- ----------

27988_wk.12_18 ---------- ---------- ---------- ---------- ----------

27988_wk.12_2 ---------- ---------- ---------- ---------- ----------

27988_wk.12_20 ---------- ---------- ---------- ---------- ----------

27988_wk.12_21 ---------- ---------- ---------- ---------- ----------

27988_wk.12_22 ---------- ---------- ---------- ---------- ----------

27988_wk.12_23 ---------- ---------? ---------- ---------- ----------

27988_wk.12_25 ---------- ---------- ---------- ---------- ----------

27988_wk.12_26 ---------- -L-------- ---------- ---------- ----------

27988_wk.12_27 ---------- ---------- ---------- ---------- ----------

27988_wk.12_28 ---------- ---------- ---------- ---------- ----------

27988_wk.12_29 ---------- ---------- ---------- ---------- ----------

27988_wk.12_3 ---------- ---------- ---------- ---------- ----------

27988_wk.12_30 ---------- ---------- ---------- ---------- ----------

27988_wk.12_4 ---------- ---------- ---------- ---------- ----------

27988_wk.12_6 ---------- ---------- ---------- ---------- ----------

27988_wk.12_7 ---------- -L-------- ---------- ---------- ----------

27988_wk.12_8 ---------- ---------- ---------- ---------- ----------

27988_wk.12_9 ---------- ---------- ---------- ---------- ----------

27988_wk.13_1 ---------- ---------- ---------- ---------- ----------

27988_wk.13_10 ---------- ---------- ---------- ---------- ----------

27988_wk.13_11 ---------- ---------- ---------- ---------- ----------

27988_wk.13_12 ---------- ---------- ---------- ---------- ----------

27988_wk.13_13 ---------- ---------- ---------- ---------- ----------

27988_wk.13_14 ---------- ---------- ---------- ---------- --E-------

27988_wk.13_15 ---------- ---------- ---------- ---------- ----------

27988_wk.13_16 ---------- ---------- ---------- ---------- ----------

27988_wk.13_17 ---------- ---------- ---------- ---------- ----------

27988_wk.13_18 ---------- ---------- ---------- ---------- ----------

27988_wk.13_19 ---------- ---------- ---------- ---------- ----------

27988_wk.13_2 ---------- ---------- ---------- ---------- ----------

27988_wk.13_20 ---------- ---------- ---------- ---------- ----------

27988_wk.13_21 ---------- ---------- ---------- ---------- ----------

27988_wk.13_22 ---------- ---------- ---------- ---------- ----------

27988_wk.13_23 ---------- ---------- ---------- ---------- ----------

27988_wk.13_24 ---------- ---------- ---------- ---------- ----------

27988_wk.13_25 ---------- ---------- ---------- ---------- ----------

27988_wk.13_26 ---------- ---------- ---------- ---------- ----------

27988_wk.13_27 ---------- ---------- ---------- ------R--- ----------

27988_wk.13_28 ---------- ---------- ---------- ---------- ----------

27988_wk.13_29 ---------- ---------- ---------- ---------- ----------

27988_wk.13_30 ---------- ---------- ---------- ---------- ----------

27988_wk.13_3 ---------- ---------- ---------- ---------- ----------

27988_wk.13_5 ---------- ---------- ---------- ---------- ----------

27988_wk.13_6 ---------- ---------- ---------- ---------- ----------

27988_wk.13_7 ---------- ---------- ---------- ---------- ----------

27988_wk.13_8 ---------- ---------- ---------- ---------- ----------

Consensus KQIVQRHLVV ETGTAETMPK TSRPTAPSSG RGGNYPVQQI GGNYVHLPLS 150

27988_wk.12_1 ---------- ---------- ---------- ---------- ----------

27988_wk.12_10 ---------- ---------- ---------- ---------- -D--------

27988_wk.12_12 ---------- ------A--- ---------- ---------- ----------

27988_wk.12_13 ---------- ---------- ---------- ---------- ----------

27988_wk.12_14 ---------- ---------- ---------- ---------- ----------

27988_wk.12_15 ---------- ---------- ---------- ---------- ----------

27988_wk.12_16 ---------- ---------- ---------- ---------- ----------

27988_wk.12_17 ---------- ---------- ---------- ---------- ----------

27988_wk.12_18 ---------- ---------- ---------- ---------- ----------

27988_wk.12_2 ---------- ---------- ---------- ---------- ----------

27988_wk.12_20 ---------- ---------- ---------- ---------- ------Q---

27988_wk.12_21 ---------- ---------- ---------- ---------- ----------

27988_wk.12_22 ---------- ---------- ---------- ---------- ----------

27988_wk.12_23 ---------- ---------- ---------- ---------- ----------

27988_wk.12_25 ------R--- ---------- ---------- ---------- ----------

27988_wk.12_26 ---------- ---------- ---------- ---------- ----------

27988_wk.12_27 ---------- ---------- ---------- ---------- ----------

27988_wk.12_28 ---------- ---------- ---------- ---------- ----------

27988_wk.12_29 ---------- ---------- ---------- ---------- ----------

27988_wk.12_3 ---------- ---------- --------G- ---------- ----------

27988_wk.12_30 ---------- ---------- ---------- ---------- ----------

27988_wk.12_4 ---------- ---------- ---------- ---------- --D-------

27988_wk.12_6 ---------- ---------- ---------- ---------- ----------

27988_wk.12_7 ---------- ---------- ---------- ---------- ----------

27988_wk.12_8 ---------- ---------- ---------- ---------- ----------

27988_wk.12_9 ---------- ---------- ---------- ---------- ----------

27988_wk.13_1 ---------- ---------- ---------- ---------- ----------

27988_wk.13_10 ---------- ---------- ---------- ---------- ----------

27988_wk.13_11 ---------- ---------- ---------- ---------- ----------

27988_wk.13_12 ---------- -------LL- ---------- ---------- ----------

27988_wk.13_13 ---------- ---------- ---------- ---------- ----------

27988_wk.13_14 ---------- ---------- ---------- ---------- ----------

27988_wk.13_15 ---------- ---------- ---------- ---------- ----------

27988_wk.13_16 ---------- ---------- ---------- ---------- ----------

27988_wk.13_17 ---------- ---------- ---------- ---------- ----------

27988_wk.13_18 ---------- ---------- ---------S ---------- ----------

27988_wk.13_19 ---------- ---------- ---------- ---------- ----------

27988_wk.13_2 ---------- ---------- ---------- ---------- ----------

27988_wk.13_20 ---------- ---------- ---------- ---------- ----------

27988_wk.13_21 ---------- ---------- ---------- ---------- ----------

27988_wk.13_22 ---------- ---------- ---------- ---------- ----------

27988_wk.13_23 ---------- ---------- ---------- ---------- ----------

27988_wk.13_24 ---------- ---------- ---------- ---------- ----------

27988_wk.13_25 ---------- ---------- ---------- ---------- ----------

27988_wk.13_26 ---------- ---------- ---------- ---------- ----------

27988_wk.13_27 ---------- ---------- ---------- ---------- ----------

27988_wk.13_28 ---------- ---------- ---------- ---------- ----------

27988_wk.13_29 ---------- ---------- ---------- ---------- ----------

27988_wk.13_30 ---------- ---------- ---------- ---------- ----------

27988_wk.13_3 ---------- ---------- ---------- ---------- ----------

27988_wk.13_5 ---------- ---------- ---------- ---------- ----------

27988_wk.13_6 ---------- ---------- ---------- ---------- ----------

27988_wk.13_7 ---------- ---------- ---------- ---------- ----------

27988_wk.13_8 ---------- ---------- ---------- ---------- ----------

**28889**

Consensus MGARNSVLSG KKADELEKIR LRPNGKKKYM LKHVVWAANE LDRFGLAESL 50

28889_wk.12_1 ---------- ---------- ---------- ---------- ----------

28889_wk.12_10 ---------- ---------- ---G------ ---------- ----------

28889_wk.12_11 ---------- ---------- ---------- ---------- ----------

28889_wk.12_12 ---------- -E-------- ---G------ ---------- ----------

28889_wk.12_13 ----D----- ---------- ---------- ---------- ----------

28889_wk.12_14 ---------- ---------- ---------- ---------- ----------

28889_wk.12_15 ---------- ---------- ---------- ---------- ----------

28889_wk.12_16 ---------- ---------- ---------- ---------- ----------

28889_wk.12_17 ---------- ---------- ---------- ---------- ----------

28889_wk.12_18 ---------- ---------- ---------- ---------- ----------

28889_wk.12_19 ---------- ---------- ---------- ---------- ----------

28889_wk.12_2 ---------- ---------- ---------- ---------- ----------

28889_wk.12_20 ---------- ---------- ---------- ---------- ----------

28889_wk.12_21 ---------- ---------- P--------- ---------- ----------

28889_wk.12_22 ---------- ---------- -------R-- ---------- ----------

28889_wk.12_24 ---------- ---------- ---G------ ---------- ----------

28889_wk.12_25 ---------- ---------- ---------- ---------- ----------

28889_wk.12_26 ---------- ---------- ---------- ---------- ----------

28889_wk.12_27 ---------- ---------- -------R-- ---------- ----------

28889_wk.12_28 ---------- ---------- ---------- ---------- ----------

28889_wk.12_29 ---------- ---------- ---------- ---------- ----------

28889_wk.12_3 ---------- ---------- ---------- --R------- ------T---

28889_wk.12_30 -----P---- ---------- ---------- -------T-- ----------

28889_wk.12_4 ---------- ---------- ---------- ---------- ----------

28889_wk.12_5 ---------- ---------- ---------- ---------- ----------

28889_wk.12_6 ---------- ---------- ---------- ---------- ----------

28889_wk.12_7 ---------- ---------- ---------- ---------- ----------

28889_wk.12_8 ---------- --------V- ---------- ---------- ----------

28889_wk.12_9 ---------- ---------- ---------- ---------- ----------

28889_wk.13_10 ---------- ---------- --H------- ---------- ----------

28889_wk.13_11 ---------- ---------- ---------- ---------- ----------

28889_wk.13_12 ---------- ---------- ---------- ---------- ----------

28889_wk.13_13 ---------- ---------- ---------- ---------- ----------

28889_wk.13_15 ---------- ---------- ---------- ---------- ----------

28889_wk.13_17 ---------- ---------- ---------- ---------- ----------

28889_wk.13_18 ---------- ---------- ---------- ---------- ----------

28889_wk.13_19 ---------- ---------- ---------- ---------- ----------

28889_wk.13_2 ?--------- ---------- ---------- ---------- ----------

28889_wk.13_21 ---------- ---------- ---------- ---------- ----------

28889_wk.13_22 ---------- ---------- ---G------ ---I------ ----------

28889_wk.13_23 ---------- ---------- ---------- ---------- ----------

28889_wk.13_25 ---------- ---------- ---------- ---------- ----------

28889_wk.13_27 ---------- ---------- ---------- ---------- ----------

28889_wk.13_28 ---------- ---------- ---------- ---------- ----------

28889_wk.13_29 ---------- ---------- ---------- ---------- ----------

28889_wk.13_3 ---------- ---------- ---------- ---------- ----------

28889_wk.13_30 ---------- ---------- ---------- ---------- ----------

28889_wk.13_5 ---------- ----A----- ---------- ---------- ----------

28889_wk.13_7 ---------- ---------- ---------- ---------- ----------

28889_wk.13_9 ---------- ---------- ---------- ---------- ----------

Consensus LENKEGCQKI LSVLAPLVPT GSENLKSLYN TVCVIWCIHA EEKVKHTEEA 100

28889_wk.12_1 ---------- ---------- ---------- ---------- ----------

28889_wk.12_10 ---------- ---------- ---------- ---------- ----------

28889_wk.12_11 ---------- ---------- ---------- ---------- ----------

28889_wk.12_12 ---------- ---------- ---------- ---------- ----------

28889_wk.12_13 ---------- ---------- -P-------- ---------- ----------

28889_wk.12_14 ---------- ---------- ---------- ---------- ----------

28889_wk.12_15 ---------- ---------- ---------- ---------- ----------

28889_wk.12_16 ---------- ---------- ---------- ---------- ----------

28889_wk.12_17 ---------- ---------- ---------- ---------- ----------

28889_wk.12_18 ---------- ---------- ---------- ---------- ----------

28889_wk.12_19 ---------- ---------- ---------- -A-------- ----------

28889_wk.12_2 ---------- ---------- ---------- ---------- ----------

28889_wk.12_20 --------R- ---------- ---------- ---------- ----------

28889_wk.12_21 ---------- ---------- ---------- ---------- ----------

28889_wk.12_22 ---------- ---------- ---------- ---------- ----------

28889_wk.12_24 ---------- ---------- ---------- ---------- ----------

28889_wk.12_25 ---------? ---------- ---------- ---------- ----------

28889_wk.12_26 ---------- ---------- ---------- ---------- ----------

28889_wk.12_27 ---------- ---------- ---------- ---------- ----------

28889_wk.12_28 ---------- ---------- ---------- ---------- ----------

28889_wk.12_29 ---------- ---------- ---------- ---------- ----------

28889_wk.12_3 ---------- ---------- ---------- ---------- ----------

28889_wk.12_30 ---------- P--------- ---------- ---------- ----------

28889_wk.12_4 ---------- ---------- ---------- ---------- ----------

28889_wk.12_5 ---------- ---------- ---------- ---------- ----------

28889_wk.12_6 ---------- ---------- ---------- ---------- ----------

28889_wk.12_7 ---------- ---------- ---------- ---------- ----------

28889_wk.12_8 ---------- ---------- ---------- ---------- ----------

28889_wk.12_9 ---R------ ---------- ---------- ---------- ----------

28889_wk.13_10 ---------- ---------- -P-------- ---------- ----------

28889_wk.13_11 ---------- ---------- ---------- ---------- ----------

28889_wk.13_12 ---------- ---------- ---------- ---------- ----------

28889_wk.13_13 ---------- ---------- ---------- ---------- ----------

28889_wk.13_15 ---------- ---------- ---------- ---------- ----------

28889_wk.13_17 ---------- ---------- ---------- ---------- ----------

28889_wk.13_18 ---------- ---------- ---------- ---------- ----------

28889_wk.13_19 ---------- ---------- ---------- ---------- ----------

28889_wk.13_2 ---------- ---------- ---------- ---------- ----------

28889_wk.13_21 ---------- ---------- ---------- ---------- ----------

28889_wk.13_22 ---------- ---------- ---------- ---------- ----------

28889_wk.13_23 ---------- ---------- ---------- ---------- ----------

28889_wk.13_25 ---------- ---------- ---------- ---------- ----------

28889_wk.13_27 ---------- ---------- ---------- ---------- ----------

28889_wk.13_28 ---------- ---------- ---------- ---------- ----------

28889_wk.13_29 ---------- ---------- ---------- ---------- ----------

28889_wk.13_3 ---------- ---------- ---------- ---------- ----------

28889_wk.13_30 ---------- ---------- ---------- ---------- ----------

28889_wk.13_5 ---------- ---------- ---------- ---------- ----------

28889_wk.13_7 ---------- ---------- ---------- ---------- ----------

28889_wk.13_9 ---------- ---------- ---------- ---------- ----------

Consensus KQIVQRHLVV ETGTAETMPK TSRPTAPSSG RGGNYPVQQI GGNYVHLPLS 150

28889_wk.12_1 ---------- ------A--- ---------- ---------- ----------

28889_wk.12_10 ---------- ---------- ---------- ---------- ----------

28889_wk.12_11 ---------- ---------- ---------- ---------- ----------

28889_wk.12_12 ---------- ---------- ---------- ---------- ----------

28889_wk.12_13 ---------- ---------- ---------- ---------- ----------

28889_wk.12_14 ---------- ---------- ---------- ---------- ----------

28889_wk.12_15 ---------- ---------- ---------- ---------- ----------

28889_wk.12_16 ---------- ---------- ---------- --------LT ----------

28889_wk.12_17 ---------- ---------- ---------- ---------- ----------

28889_wk.12_18 ---------- ---------- ---------- ---------- ----------

28889_wk.12_19 ---------- ---------- ---------- ---------- ----------

28889_wk.12_2 ---------- ---------- ---------- ---------- ----------

28889_wk.12_20 ---------- ---------- ---------- ---------- ----------

28889_wk.12_21 ---------- ---------- ---------- ---------- ---------G

28889_wk.12_22 ---------- ---------- ---------- ---------- ----------

28889_wk.12_24 ---------- ---------- ---------- ---------- ----------

28889_wk.12_25 ---------- ---------- ---------- ---------- ----------

28889_wk.12_26 ---------- ---------- ---------- ---------- ----------

28889_wk.12_27 ---------- ---------- ---------- ---------- ----------

28889_wk.12_28 ---------- ---------- ---------- ---------- ----------

28889_wk.12_29 ---------- ---------- P--------- ---------- ----------

28889_wk.12_3 ---------- G--------- ---------- ---------- ----------

28889_wk.12_30 ---------- ---------- ---------- ---------- ----------

28889_wk.12_4 ---------- ---------- ---------- ---------- ----------

28889_wk.12_5 ---------- ---------- ---------- ---------- ------Q---

28889_wk.12_6 ---------- ---------- ---------- ---------- ----------

28889_wk.12_7 ---------- ---------- ---------- ---------- ----------

28889_wk.12_8 ---------- ---------- ---------- ---------- ----------

28889_wk.12_9 ---------- ---------- ---------- ---------- ----------

28889_wk.13_10 ---------- ---------- --------G- ---------- ----------

28889_wk.13_11 ---------- ---------- ---------- ---------- ----------

28889_wk.13_12 ---------- ---------- ---------- ---------- ----------

28889_wk.13_13 ---------- ---------- ---------- ---------- ----------

28889_wk.13_15 ---------- ---------- ---------- ---------- ----------

28889_wk.13_17 ---------- ---------- ---------- ---------- ----------

28889_wk.13_18 ---------- ---------- ---------- ---------- ----------

28889_wk.13_19 ---------- ---------- ---------- ---------- ----------

28889_wk.13_2 ---------- ---------- ---------- G--------- ----------

28889_wk.13_21 ---------- ---------- ---------- ---------- ----------

28889_wk.13_22 ---------- ---------- ---------- ---------- ----------

28889_wk.13_23 ---------- ---------- ---------- ---------- ----------

28889_wk.13_25 ---------- ---------- ---------- ---------- ----------

28889_wk.13_27 ---------- ---------- ---------- ---------- ----------

28889_wk.13_28 ---------- ---------- ---------- ---------- ----------

28889_wk.13_29 ---------- ---------- ---------- ---------- ----------

28889_wk.13_3 ---------- ---------- ---------- ---------- ----------

28889_wk.13_30 ---------- ---------- ---------- ---------- ----------

28889_wk.13_5 ---------- ---------- ---------- ---------- ----------

28889_wk.13_7 ---------- ---------- ---------- ---------- ----------

28889_wk.13_9 ---------- ---------- ---------- ---------- ----------
